# Symptomatic spinal metastasis: A systematic literature review of the preoperative prognostic factors for survival, neurological, functional and quality of life in surgically treated patients and methodological recommendations for prognostic studies

**DOI:** 10.1371/journal.pone.0171507

**Published:** 2017-02-22

**Authors:** Anick Nater, Allan R. Martin, Arjun Sahgal, David Choi, Michael G. Fehlings

**Affiliations:** 1 Department of Surgery, Division of Neurosurgery, University of Toronto, Toronto, Ontario, Canada; 2 Department of Radiation Oncology, Sunnybrook Odette Cancer Centre, Toronto, Canada; 3 Department of Neurosurgery, The National Hospital for Neurology and Neurosurgery, and Institute of Neurology, University College London, London, United Kingdom; 4 Department of Neurosurgery, Toronto Western Hospital, University Health Network, Toronto, Ontario, Canada; Taipei Medical University, TAIWAN

## Abstract

**Purpose:**

While several clinical prediction rules (CPRs) of survival exist for patients with symptomatic spinal metastasis (SSM), these have variable prognostic ability and there is no recognized CPR for health related quality of life (HRQoL). We undertook a critical appraisal of the literature to identify key preoperative prognostic factors of clinical outcomes in patients with SSM who were treated surgically. The results of this study could be used to modify existing or develop new CPRs.

**Methods:**

Seven electronic databases were searched (1990–2015), without language restriction, to identify studies that performed multivariate analysis of preoperative predictors of survival, neurological, functional and HRQoL outcomes in surgical patients with SSM. Individual studies were assessed for class of evidence. The strength of the overall body of evidence was evaluated using GRADE for each predictor.

**Results:**

Among 4,818 unique citations, 17 were included; all were in English, rated Class III and focused on survival, revealing a total of 46 predictors. The strength of the overall body of evidence was *very low* for 39 and *low* for 7 predictors. Due to considerable heterogeneity in patient samples and prognostic factors investigated as well as several methodological issues, our results had a moderately high risk of bias and were difficult to interpret.

**Conclusions:**

The quality of evidence for predictors of survival was, at best, *low*. We failed to identify studies that evaluated preoperative prognostic factors for neurological, functional, or HRQoL outcomes in surgical patients with SSM. We formulated methodological recommendations for prognostic studies to promote acquiring high-quality evidence to better estimate predictor effect sizes to improve patient education, surgical decision-making and development of CPRs.

## Introduction

Symptomatic spinal metastasis (SSM) afflicts up to 10% of cancer patients[1–3], of which approximately 10% are surgically managed.[4] Given that over 14 million Americans lived with a diagnosis of cancer in 2014 and almost 19 million are expected to do so by 2024[5], the number of cancer survivors expected to undergo surgery for SSM will increase by approximately 36% over the next 10 years.

Since the randomized controlled trial conducted by Patchell et al.[6] showing that surgery followed by radiotherapy provided superior neurologic outcomes compared to radiotherapy alone in patients suffering from a single cervical or thoracic SSM with a life expectancy of ≥ 3 months, this life expectancy threshold has been widely adopted in decision-making for surgical treatment.[7–9] However, clinicians and surgeons tend to estimate survival in patients with advanced cancer inaccurately.[10–13] Also, although several studies reported that surgical intervention improved health related quality of life (HRQoL)[6, 9, 14–20], SSM treated with surgery is the most costly skeletal-related event in patients with cancer.[21]

Clinical prediction rules (CPRs), which combine various clinical factors from an individual with a given health state and provide an estimate of the risk of experiencing a specific endpoint within a certain period[22], may allow physicians to make more precise clinical estimates and thus assist therapeutic decision-making and counselling.[22, 23] Although several CPRs of survival have been elaborated, we are not aware of any CPR for HRQoL for SSM patients. Also, current CPRs of survival have variable prognostic ability.[24–26] This may be due to differences between patient samples that were used to generate and conduct prognostic value assessment. For instance, Bartels et al.[27] created a CPR of survival based on a cohort of patients who received radiotherapy. In their most recent external validation study[28], misspecification of their model was attributed to the surgical patient subgroup.

The majority of published series assessed preoperative predictors of survival rather than HRQoL. We conducted a systematic review to ascertain the preoperative prognostic factors for 1) survival, 2) neurologic status, 3) functional status, and 4) HRQoL in surgical SSM patients. We also appraised the methodology and reporting of prognostic studies that met our eligibility criteria. The results of this study could not only be used to modify existing CPRs of survival to improve their prognostic value, but also to improve the theoretical framework to develop new CPRs for survival and HRQoL outcomes specific to surgical SSM patients.

## Methods

This systematic review and best-evidence synthesis was conducted and reported in accordance with the Preferred Reporting Items for Systematic Reviews and Meta-Analyses (PRISMA) guidelines[29]. In compliance with the guidelines, our systematic review protocol was registered with the International Prospective Register of Systematic Reviews (PROSPERO)[30] on June 24^th^, 2015 and was last updated on July 12^th^, 2016 (registration number CRD42015023831).

### Literature search

In adult patients who underwent surgery for SSM, we sought to answer the following four key questions (KQs): What are the preoperative clinical factors associated with postoperative (1) survival; (2) neurologic status, such as muscle power on the Medical Research Council (MRC) scale for testing muscle strength, neurologic outcome measures (e.g. American Spinal Injury Association (ASIA) or Frankel grade) or autonomic functions (bladder / bowel control); (3) functional status, in terms of ambulatory status, functional outcome measures, such as functional independence measure (FIM), Barthel index, Eastern Cooperative Oncology Group (ECOG) or Karnofsky performance status (KPS); and (4) HRQoL, in terms of score on any HRQoL measure, such as short form health survey (SF-36), EuroQol 5 dimensions (EQ-5D) or Oswestry Disability Index (ODI)?

The electronic databases MEDLINE, MEDLINE in Process, Embase, Web of Science, CINAHL, Cochrane Central Register of Controlled Trials, and Scopus were systematically searched for studies performed in humans from January 1, 1990 to December 31, 2015 with no language restrictions applied. The search strategies were developed in consultation with information specialists at the University Health Network Health Sciences Libraries. S1 Table presents our complete search strategies. The reference lists of studies meeting the eligibility criteria and relevant review papers were manually screened for additional studies.

### Eligibility criteria

Citations were screened for eligibility by following *a priori* determined inclusion and exclusion criteria (Table 1). Original studies with an identifiable surgical treatment arm or surgical cohort of at least 30 patients, who underwent *de novo* spinal surgery for a single symptomatic metastatic spinal lesion, with a postoperative follow-up of at least 6 months, published in peer-reviewed journals included in Ulrichsweb[31] at the time of publication, describing and reporting both the preoperative prognostic clinical factors assessed and the univariate and multivariate analyses conducted, were considered for inclusion. Studies that included surgical/postoperative predictors in their multivariate analyses, patients < 18 years old, patients operated for recurrent SSM or primary spinal tumor were excluded.

**Table 1:** Inclusion and exclusion criteria.

	Inclusion	Exclusion
**Patient**	De novo surgically treated adults MESCC patients included in a surgical series of at least 30 patients published from January 1, 1990 to December 31, 2015	MESCC due to trauma, infection, stenosis, degenerative changes, primary CNS or vertebral tumor Pediatric (< 18 years)
**Intervention**	Surgical treatment^1^ performed for at least one of the following indications: Intractable pain resulting from a symptomatic MESCC lesion Spinal instability: imminent or overt Onset or progression of neurologic deficits, i.e. sensorimotor or autonomic	All non-surgical treatments (hormonotherapy, immunotherapy, chemotherapy, corticosteroid, and radiotherapy, including conventional external beam radiotherapy, intensity-modulated radiotherapy, stereotactic radiosurgery / stereotactic body radiation therapy, stereotactic radiotherapy and systemic application of radioisotopes) used alone Surgery for recurrent MESCC lesion
**Study design**	Original series From all languages and at the time of publication, published in peer-reviewed journals between January 1, 1990 to December 31, 2015 Prospective and retrospective studies with a follow-up of at least six months With an identifiable surgical treatment arm or surgical cohort of at least 30 patients Providing adequate description of pre-operative factors^2^ With adequate description of the (1) preoperative predictive clinical factors assessed and (2) univariate and multivariate analyses conducted With the results of univariate and multivariate analyses clearly reported Studies that used the same data were individually included as long as they satisfy our eligibility criteria Studies validating or examining the accuracy of existing scoring systems were included if (1) they met the eligibility criteria, (2) patient sample was different from the one used to develop the scoring system (3) and the predictive value of the preoperative clinical factors constituting the scoring system were individually assessed and clearly reported.	Animal or biomechanical studies Opinions Commentaries Editorials Conference proceedings Systematic reviews Meta-analyses Studies that involved multivariate analysis that included surgical or postoperative factors as predictors
**Outcome**	Survival Neurologic outcomes muscle power sphincter dysfunction, i.e. bladder and bowel control sexual dysfunction ASIA or Frankel score Functional outcomes Karnofsky / ECOG performance status Ambulatory status Quality of life Score on any given metrics or instruments assessing health related quality of life	

^1^ Spinal surgery refers to a *de novo* surgical treatment involving any open or minimally invasive spinal interventions, including vertebroplasty and kyphoplasty, to achieve partial or complete spinal decompression and/or mechanical stabilization, with or without instrumentation

^2^ Potential pre-operative predictive factors include clinical features such as sex, age, ethnicity, body mass index (BMI), smoking status, histologic type or site of the primary tumor, biomarkers, treatment received for primary and/or spinal metastasis, performance status score and SF-36. Potential surgical predictive factors include type of surgery and extent of tumor resection, length of operation and blood loss.

**ASIA**: American Spinal Injury Association; **ECOG**: Eastern Cooperative Oncology Group; **MESCC**: metastatic epidural spinal cord compression

### Screening and selection

All duplicates were removed using EndNote X4 followed by manual elimination. Two authors (AN and ARM) independently (1) screened the titles and abstracts to identify potential eligible studies to undergo full-text assessment and then (2) reviewed the selected full-text articles for final inclusion. Discrepancies between the two reviewers were resolved by consensus agreement; persisting disagreements were settled by consulting the senior author (MGF).

### Data extraction and synthesis

The following data were extracted by AN and then checked by ARM: 1) first author and publication date; 2) publication language; 3) study design; 4) purpose; 5) patient sample and characteristics, with relevant inclusion and exclusion criteria; 6) preoperative predictors 7) outcome assessed; 8) postoperative follow-up characteristics, including length, rate, and information about how missing data were handled; 9) methodology, including details related to predictors’ selection, type of univariate and multivariate analyses conducted, multivariate modeling process and assumption(s) testing; and 10) univariate and multivariate estimates, including reported odds / hazard ratios and confidence intervals. Unless otherwise specified, a p-value < 0.05 was considered statistically significant.

### Critical appraisal of the literature

We are not aware of any consensus regarding a standardized approach for assessing the quality of prognostic studies.

### Risk of bias in individual studies

AN and ARM independently assessed the risk of bias of individual articles (Class I to IV) using the method described by Skelly et al.[32, 33] for prognostic studies (S2 Table). The final class-of-evidence rating was assigned following consensus agreement.

### Risk of bias across studies: Overall quality of evidence

Once all articles were individually evaluated, the strength of the overall body of evidence with respect to each predictor was allocated using the approach developed by the Grading of Recommendation Assessment, Development and Evaluation (GRADE) Working Group.[34] The *baseline* strength of the overall body of evidence was assigned “High” if the majority of the studies were Class I or II and “Low” if the majority of the studies were Class III or IV. The strength could then be downgraded by one or two levels based on the risk of bias, consistency, directness, precision and publication bias. Alternatively, the strength could be upgraded by one or two levels if the effect was large, there was evidence of a dose response gradient or all plausible confounders would either reduce a demonstrated effect or would suggest a spurious effect when the results showed no effect. The *final* strength of the overall body of evidence for each predictor was classified as High, Moderate, Low or Very Low and expresses our confidence that the evidence reflects the true effect and the likelihood of further research to change our confidence in the latter estimate of effect (S3 Table). Overall, this method adheres to the general principles described by Hayden et al.[35] for assessing the quality of prognostic studies in systematic reviews.

## Results

**Fig 1:**
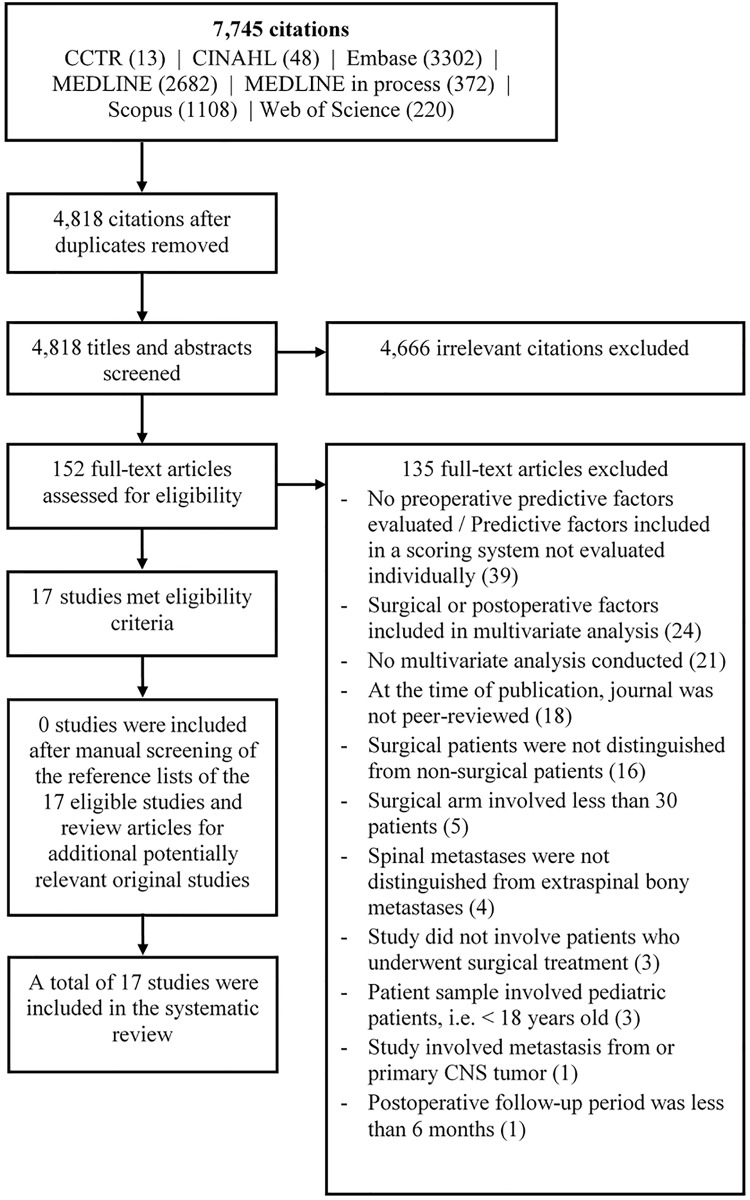
Study selection process.

The search yielded 4,818 unique citations, of which the title and abstract were screened, leading to the selection of 152 articles for full-text review. Among these, a total of 135 studies were excluded for one of the following reasons: preoperative prognostic factors were not evaluated or were assessed as part of a scoring system and not evaluated individually; multivariate analysis was not conducted; multivariate analysis included surgical and/or postoperative factors as predictors; the journal was not peer-reviewed at the time of publication on Ulrichsweb[31]; surgical patients were not evaluated separately from non-surgical patients; the study included less than 30 surgical patients; spinal metastases were not distinguished from extraspinal bony metastases; patient sample included patients < 18 years of age; the study involved metastasis from primary central nervous system tumors; postoperative follow-up was less than six months. No additional studies were added after manually checking reference lists (Fig 1).

All 17 articles meeting our eligibility criteria were published in English and addressed KQ1, i.e. the preoperative clinical factors associated with survival in surgical SSM patients. There were six additional studies that examined the clinical prognostic factors of functional status (KQ3) in terms of the ability to walk[36–41] or regaining the ability to walk[37] postoperatively and one study that isolated key predictors of survival (KQ1) and HRQoL (KQ4) using the postoperative EQ-5D score as the dependent variable[42]. However, these studies included surgical and/or postoperative factors in their multivariate analysis, leading to their exclusion from this review.

### Survival

All 17 studies investigated the prediction of survival, pursuing one or more of the following aims: to evaluate (1) predictors of survival; (2) predictors of survival and propose a prognostic model or a scoring system to predict survival; (3) the prognostic value of parameters included in an existing scoring system that predict survival. Ten studies examined aim #1[43–52], including four that analyzed a heterogeneous population of primary tumor types[43–45, 50], one study focused on patients older than 60 years of age[46], and the remainder investigated specific primary malignancy types including breast cancer[48], hepatocellular carcinoma (HCC)[52], prostate cancer[49] or non-small cell lung cancer (NSCLC)[51] and unknown primary[47]. Four studies addressed aim #2[41, 53–55], including one that involved a heterogeneous primary tumor type[53], prostate cancer[54], lung cancer[55] or NSCLC[41]. Four studies explored aim #3[24, 52, 56, 57], with three involving heterogeneous primary tumor type[24, 56, 57] and one focused on HCC[52].

### Risk of bias of individual studies

Prospective prognostic studies meeting the following criteria for a good-quality cohort study are considered Class I evidence: (1) patients were followed for sufficient periods in order that outcomes could occur, (2) follow-up rate was ≥ 80%, (3) patients were at similar point in the course of their disease, and (4) the study accounted for other prognostic factors (S2 Table). Although this review included three prospective studies[24, 47, 56], they were considered as Class III evidence due to violation of two of the criteria for good-quality cohort studies: follow-up period and drop-out rate were not clearly specified. The remaining 14 retrospective studies were also considered Class III due to violation of at least one of the criteria for good-quality studies.

### Results of individual studies

A summary of the 17 studies is presented in Table 2. Numerous preoperative clinical factors were reported to negatively impact survival when all sites of primary tumor were considered: primary tumor type (e.g. lung, colon)[43, 45, 56], radioresistant primary tumor[50], primary tumor Tomita Grade III (modified Tomita classification[57] and original classification)[24], primary tumor Tomita Grade II and III (original classification)[53], poor prognosis of primary tumor (based of median survival < 20 months[44]), presence of visceral metastasis[24, 53, 56, 57], KPS < 80%[56, 57], presence of neurologic deficit (e.g. palsy)[43, 44], presence of non-symptomatic spinal metastasis[53, 56], incapacity to walk independently or with a walking aid[50], Charlson comorbidity index score (CCIS) ≥ 2[50], older age[43], male sex[43], presence of pain[44], and ASIA score B or C[45]; and a score of 9–12 points on the original Tokuhashi scoring system and primary tumor Tomita Grade III (original classification)[46] in patients ≥ 60 years old.

**Table 2:** Evidentiary table of 17 studies identifying preoperative predictive factors of survival in surgical SSM patients.

1^st^ Author, Year, Study design (Class of evidence)	Study sample characteristics†	Statistical method Univariate; Multivariate	Predictive factors assessed‡	Results (HR; 95% CI; p-value)
Aizenberg, 2012, Prospective (III)	Single center June 1993 to February 2007 n = 51 Unknown primary tumor Median age: 59.9 16 F: 35 M	Cox PH model for both uni- and multivariate Variables with p < 0.15 in univariate analysis were included in multivariate analysis and tested through a backward stepwise selection	**Spinal location** (cervical vs. other location)	Multivariate: HR: NR. *cervical*: MS: 6.4 mo; 95% CI: 1.1–11.7; *other location*: MS: 11.8 mo; 95% CI: 4.5–19.11; p = 0.01
			**Frankel score** (A, B, C vs. D, E)	Multivariate: HR: NR. *A*, *B*, *C*: MS: 2.7 mo; 95% CI: 0–6.3; *D*, *E*: MS: 11.8 mo; 95% CI: 6–17.8; p = 0.029
			**Extraspinal disease at presentation** (present vs. absent)	Multivariate: HR: NR. *present*: MS: 6.4 mo; 95% CI: 2.6–10.2; *absent*: 18.1 mo; 95% CI: 10.1–26.1; p = 0.041
			Other spine disease (present vs. absent)	Multivariate: HR: NR. *present*: MS: 12.7 mo; 95% CI: 0.6–24.8; *absent*: MS: 8.7 mo; 95% CI: 0–18.3; p > 0.05
			Timing of surgery, i.e. from presentation to surgery (≥ 2.6 vs. < 2.6 mo)	Multivariate: HR: NR *≥ 2*.*6*: MS: 12.7 mo; 95% CI: 1–24.5; *< 2*.*6*: MS: 6.8 mo; 95% CI: 2.9–10.7; p > 0.05
			Extend of resection (GTR vs. STR, surgeon’s estimate at surgery)	Univariate: HR: NR. *GTR*: MS: 8.1 mo; 95% CI: 0–18.5; *STR*: 6.4 mo; 95% CI: 0–13.2; p = 0.18
			Lymph node involved at presentation (involved vs. not)	Univariate: HR: NR. *involved*: MS: 8.1 mo; 95% CI: 0–17.2; *not involved*: 8.7 mo; 95% CI: 0–17.4; p = 0.40
			Age at surgery (> 60 vs. ≤ 60 years, based on median age)	Univariate: HR: NR. *60*: MS: 8.7 mo; 95% CI: 0–17.6; *≤ 60*: MS: 8.1 mo; 95% CI: 0–17.9; p = 0.56
			Sex (female vs. male)	Univariate: HR: NR. *Female*: MS: 5.4 mo; 95% CI: 2.9–7.8; *Male*: MS: 12.8 mo; 95% CI: 2.1–23.4; p = 0.17
			Spinal metastatic disease as initial presentation (yes vs. no)	Univariate: HR: NR. *yes*: MS: 8.7 mo; 95% CI: 1.1–16.2; *no*: MS: 8.1 mo; 95% CI: 0–25; p = 0.82
			Prior treatment to spinal metastasis (yes vs. no)	Univariate: HR: NR. *yes*: MS: 6.8 mo; 95% CI: 2.8–10.8; *no*: MS: 12.8 mo; 95% CI: 1.1–24.4; p = 0.25
Arrigo, 2011, Retrospective (III)	Single center 1999 to 2009 n = 200 All primaries, including multiple myeloma, lymphoma, plasmacytoma Average age: 58.9 78 F: 122 M	Univariate: Categorical variables: Kaplan-Meier and Log-rank test; continuous variables: Wald test Multivariate: Variables with p < 0.15 in univariate analyses or predictive significance was previously suggested by other authors were included in Cox PH analysis	**Radiosensitive primary** (yes vs. no)	*Univariate*: p < 0.0001. *Multivariate*: HR: 2.557; 95% CI: 1.672–3.912; p < 0.0001
			**Ambulatory status** (yes ± walker, vs. no)	*Univariate*: p < 0.0001. *Multivariate*: HR: 2.355; 95% CI: 1.517–3.658; p = 0.0001
			**Charlson comorbidity index score** (0, 1 vs. ≥ 2)	*Univariate*: p = 0.0058. *Multivariate*: HR: 2.955; 95% CI: 1.341–6.512, p = 0.0072
			Age at time of surgery (years)	*Univariate*: p = 0.9936. *Multivariate*: HR: 1.001; 95% CI: 0.986–1.016; p = 0.8956
			Cervical spinal location (*category not specified*)	*Univariate*: NR. *Multivariate*: HR: 1.070; 95% CI: 0.693–1.652; p = 0.7611
			Pathological fracture (present vs. absent)	*Univariate*: p = 0.4813. *Multivariate*: HR: 1.408; 95% CI: 0.954–2.078; p = 0.0853
			Radiotherapy to surgical site given (yes vs. no)	*Univariate*: p = 0.0829. *Multivariate*: HR: 0.980; 95% CI: 0.673–1.427; p = 0.9144
			Visceral metastases (present vs. absent)	*Univariate*: p = 0.3704. *Multivariate*: HR: 1.089; 95% CI: 0.754–1.573; p = 0.6486
			Epidural compression (present vs. absent)	*Univariate*: p = 0.0916. *Multivariate*: HR: 1.201; 95% CI: 0.691–2.085; p = 0.5160
			Functional health status (independent vs. partially/fully dependent)	Univariate: p < 0.0001
			Frankel grade (E vs. C, D vs. A, B)	Univariate: p = 0.0001
			Primary tumor type (lung vs. breast vs. prostate vs. renal vs. colon vs. other)	Univariate: p = 0.0002
			ASA (ASA 2 vs. ASA 3, 4)	Univariate: p = 0.0014
			Smoker during past year (yes vs. no)	Univariate: p = 0.0043
			Urinary function (reten-tion/incontinence vs. no)	Univariate: p = 0.0843
			Sex (female vs. male)	Univariate: p = 0.2566
			Extraspinal bone metastasis (yes vs. no)	Univariate: p = 0.2719
			Number of vertebrae with metastasis present	Univariate: p = 0.3918
Bollen, 2013, Retrospective (III)	2 centers January 2001 to December 2010 n = 106 All primaries (inclusion of hematologic primaries is not specified) Mean age: 59.0 53 F: 53 M	Univariate: Kaplan-Meier and Log-rank test Multivariate: Cox PH model, backward stepwise procedure was performed using: modified primary tumor Tomita grade, visceral metastasis, and KPS	**Modified primary tumor Tomita grade** (slow vs. moderate vs. rapid)	*Univariate*: p < 0.0001. *Multivariate*: rapid vs. slow: HR: 3.1; 95% CI: 1.6–6.2; p = 0.001. mod. vs. slow: HR: 1.7; 95% CI: 0.9–3.3; p = 0.099
			**KPS** (80–100% vs. 50–70% vs. 10–40%)	*Univariate*: p = 0.169. *Multivariate*: KPS 10–40 vs. 80–100%: HR: 2.7; 95% CI: 1.1–6.6; p = 0.025. KPS 50–70 vs. 80–100%: HR: 1.3; 95% CI: 0.8–2.1; p = 0.292
			**Visceral metastases** (absent vs. present)	*Univariate*: p = 0.014. *Multivariate*: HR: 1.7; 95% CI: 1.0–2.9; p = 0.033
			Primary tumor: Tokuhashi revised	Univariate: p = 0.0001
			Primary tumor: van der Linden modified	Univariate: p = 0.0002
			Primary tumor: Bauer modified	Univariate: p = 0.0008
			Tomita visceral metastases	Univariate: p = 0.027
			Age (<65 vs. ≥ 65 years)	Univariate: p = 0.089
			Tomita bone metastases (solitary spinal ± extra-spinal vs. multiple spinal ± extra-spinal metastasis)	Univariate: p = 0.946
			Extra-spinal bone metastases (0 vs. 1–2 vs. ≥3)	Univariate: p = 0.970
			Number of spinal metastases (1 vs. 2 vs. ≥ 3)	Univariate: p = 0.860
			Frankel classification (A, B vs. C, D vs. E)	Univariate: p = 0.196
			Spinal location (C-T6 vs. T7-L)	Univariate: p = 0.163
			Sex (male vs. female)	Univariate: p = 0.159
Cahill, 2011, Retrospective (III)	2 centers 1986 to 2005 n = 379 Breast cancer Mean age: 72 379 F: 0 M	Cox PH model for uni- and multivariate analyses Variables with p < 0.10 in univariate analyses were included in multivariate analysis	**Time interval from cancer diagnosis to surgery** (year)	*Univariate*: HR: 0.98; 95% CI: 0.96–1.00; p-value: NR. *Multivariate*: HR: 0.97; 95% CI: 0.95–0.99; p-value: NR
			**Admission to hospital** (emergency room vs. no)	*Univariate*: HR: 1.41; 95% CI: 1.10–1.79; p-value: NR. *Multivariate*: HR: 1.53; 95% CI: 1.20–1.97; p-value: NR
			**Histologic grade** (poor/undifferentiated vs. well/moderately differentiated)	*Univariate*: HR: 1.51; 95% CI: 1.17–1.95; p-value: NR. *Multivariate*: HR: 1.49; 95% CI: 1.14–1.95; p-value: NR
			**Progesterone receptors** (positive vs. negative)	*Univariate*: HR: 0.48; 95% CI: 0.32–0.72; p-value: NR. *Multivariate*: HR: 0.54; 95% CI: 0.34–0.87; p-value: NR
			Estrogen receptors (positive vs. negative)	*Univariate*: HR: 0.47; 95% CI: 0.28–0.79; p-value: NR. *Multivariate*: reported not significant, no results provided
			Estrogen receptors (unknown vs. negative)	*Univariate*: HR: 0.61; 95% CI: 0.37–1.00; p-value: NR. *Multivariate*: reported not significant, no results provided
			Progesterone receptors (unknown vs. negative)	*Univariate*: HR: 0.71; 95% CI: 0.50–1.00; p-value: NR. *Multivariate*: reported not significant, no results provided
			Age (year)	Univariate: HR: 0.99; 95% CI: 0.98–1.01; p-value: NR
			Race (white vs. non-white)	Univariate: HR: 0.73; 95% CI: 0.50–1.07; p-value: NR
			Living area (rural vs. no)	Univariate: HR: 1.14; 95% CI: 0.74–1.78; p-value: NR
			Marital status (married vs. no)	Univariate: HR: 0.92; 95% CI: 0.75–1.14; p-value: NR
			Charlson comorbidity score (per unit increase)	Univariate: HR: 1.05; 95% CI: 0.97–1.14; p-value: NR
			Stage 4 disease at breast cancer diagnosis	Univariate: HR: 0.99; 95% CI: 0.73–1.34; p-value: NR
Chen, 2010, Retrospective (III)	Single center January 2001 to December 2007 n = 41 Hepatocellular carcinoma Mean age: 53.15 9 F: 32 M	Univariate: Kaplan-Meier and Log-rank test Multivariate:Variables with p < 0.05 in univariate analyses were included in the multivariate Cox PH analysis	**Serum albumin** (< 37 vs. ≥ 37 g/L)	*Univariate*: p = 0.003. *Multivariate*: HR: 0.295; 95% CI: 0.106–0.819; p = 0.019
			**Lactate dehydrogenase** (< 200 vs. ≥ 200 U/L)	*Univariate*: p = 0.044. *Multivariate*: HR: 5.626; 95% CI: 1.562–20.265; p = 0.008
			Number of spinal metastases (1 vs. ≥ 2)	*Univariate*: p = 0.020. *Multivariate*: HR: 1.679; 95% CI: 0.647–4.354; p = 0.287
			KPS (≤ 70% vs. > 70%)	*Univariate*: p = 0.004. *Multivariate*: HR: 0.819; 95% CI: 0.374–1.791; p = 0.616
			Sex (female vs. male)	Univariate: NR
			Age (year)	Univariate: NR
			Metastasis to vital organs (present vs. absent)	Univariate: NR
			Extra-spinal bone metastases (0 vs. 1–2 vs. ≥ 3)	Univariate: NR
			Frankel score (A, B vs. C, D vs. E)	Univariate: NR
			Pathologic fracture (present vs. absent)	Univariate: NR
			Aspartate aminotransferase (< 40 vs. ≥ 40 U/L)	Univariate: NR
			Alanine transaminase (< 40 vs. ≥ 40 U/L)	Univariate: NR
			Spinal levels of metastasis (cervical vs. thoracic vs. lumbar vs. sacral)	Univariate: NR
Crnalic, 2012, Retrospective (III)	Single center September 2003 to September 2010 n = 53 Hormone refractory prostate cancer Mean age: 68 0 F: 53 M	Univariate: Kaplan-Meier and using Log-rank test; Cox PH model Multivariate:All variables from univariate analyses were included in the multivariate Cox PH analysis	**KPS** (80–100% vs. 50–70%)	*Univariate*: Log-rank test: p = 0.0003; Cox: HR: 4.66; 95% CI: 1.90–11.44; p = 0.001. *Multivariate*: HR: 3.97; 95% CI: 1.57–10.04; p = 0.004
			Visceral metastasis (absent vs. present)	*Univariate*: Log-rank test: p = 0.0002; Cox: HR: 2.52; 95% CI: 1.35–4.70; p = 0.004. *Multivariate*: HR: 1.80; 95% CI: 0.93–3.46; p = 0.08
			Serum PSA (< 200 vs. ≥200 ng/ml)	*Univariate*: Log-rank test: p = 0.015; Cox: HR: 2.08; 95% CI: 1.13–3.82; p = 0.019. *Multivariate*: HR: 1.47; 95% CI: 0.79–2.75; p = 0.22
			Age (< 71 vs. ≥71)	*Univariate*: Log-rank test: p = 0.85; Cox: HR: 0.95; 95% CI: 0.55–1.65; p = 0.85. *Multivariate*: HR: 0.89; 95% CI: 0.49–1.64; 0 = 0.72
			Time interval from primary tumor diagnosis to surgery (< 36 vs. ≥36 mo)	*Univariate*: Log-rank test: p = 0.87; Cox: HR: 0.96; 95% CI: 0.55–1.67; p = 0.088. *Multivariate*: HR: 1.29; 95% CI: 0.70–2.39; p = 0.41
			Ambulatory status (ambulatory vs. non-ambulatory)	*Univariate*: Log-rank test: p = 0.31; Cox: HR: 1.50; 95% CI: 0.67–3.37; p = 0.32. *Multivariate*: HR: 0.90; 95% CI: 0.38–2.17; p = 0.82
Finkelstein, 2003, Retrospective (III)	Single center July 1991 and March 1998 n = 987 All primaries including lymphoma and myeloma Mean age: 60.3 F: M—NR	Univariate: Bivariate analysis to assess unadjusted relationship between potential predictor and survival Multivariate:Cox PH model	**Age** (year)	*Univariate*: NR. *Multivariate*: HR: 1.01; 95% CI: 1.01 to 1.02; p = 0.0001
			**Gender** (female vs. male)	*Univariate*: NR. *Multivariate*: HR: 1.48; 95% CI: 1.24 to 1.77; p = 0.0001
			**Lung** (lung vs. other)	*Univariate*: NR. *Multivariate*: HR: 3.17; 95% CI: 2.57 to 3.90; p = 0.0001
			**Melanoma** (melanoma vs. other)	*Univariate*: NR. *Multivariate*: HR: 2.71; 95% CI: 1.79 to 4.13; p = 0.0001
			**Breast** (breast vs. other)	*Univariate*: NR. *Multivariate*: HR: 2.06; 95% CI: 1.58 to 2.68; P = 0.0001
			**Stomach** (stomach vs. other)	*Univariate*: NR. *Multivariate*: HR: 3.50; 95% CI: 2.07 to 5.93; P = 0.0001
			**Colon** (colon vs. other)	*Univariate*: NR. *Multivariate*: HR: 2.06; 95% CI: 1.44–2.97; P = 0.0001
			**Prostate** (prostate vs. other)	*Univariate*: NR. *Multivariate*: HR: 1.38; 95% CI: 1.08–1.76; P = 0.0092
			**Kidney** (kidney vs. other)	*Univariate*: NR. *Multivariate*: HR: 2.26; 95% CI: 1.73–2.95; P = 0.0001
			**Neurologic deficit** (no vs. yes)	*Univariate*: NR. *Multivariate*: HR: 1.19; 95% CI: 1.01–1.41; P = 0.0377
Hosono, 2005, Retrospective (III)	3 centers 1985 to 2001 n = 165 All primaries, including myeloma Age—NR F: M—NR	Univariate: Kaplan-Meier and generalized Wilcoxon test Multivariate: Variables with p < 0.05 in univariate analyses were included in the multivariate Cox PH analysis	**Primary tumor** (favorable, i.e. > 20 mo MS, included: myeloma, thyroid cancer, renal cell cancer, breast cancer, and prostate, vs. poor prognosis, i.e.< 20 mo MS, included other primaries)	*Univariate*: p < 0.0001. *Multivariate*: HR: 2.36; 95% CI: 1.67–3.35; p < 0.001
			**Pain** (without pain vs. pain)	*Univariate*: p = 0.0283. *Multivariate*: HR: 2.53; 95% CI: 1.12 to 7.26; p = 0.0228
			**Paresis** (nonparetic vs. paretic)	*Univariate*: p = 0.0003. *Multivariate*: HR: 1.79; 95% CI: 1.11 to 2.96; p = 0.0164
			Walking status (walking without vs. walking with support or non-walking)	*Univariate*: p = 0.0283. *Multivariate*: HR: 0.97; 95% CI: 0.59 to 1.60; p = 0.889
Leithner, 2008, Prospective (III)	3 centers January 1998 to September 2006 2 analyses: (i) including myeloma (n = 69) and (ii) excluding myeloma (n = 59) All other primaries, including non-Hodgkin lymphoma Mean age: 60 32 F: 37 M	Univariate: Kaplan-Meier and Log-rank test Multivariate: Cox PH model	**Primary tumor Tomita grade** (rapid vs. moderate vs. slow)	Including myeloma: *Univariate*: p < 0.001. *Multivariate*: overall: p < 0.001. mod. vs. slow: HR: 1.07; 95% CI: 0.51–2.22; p = 0.857. rapid vs. slow: HR: 9.32; 95% CI: 3.87–22.5; p < 0.001. Excluding myeloma: *Univariate*: p < 0.001. *Multivariate*: overall: p < 0.001. mod. vs. slow: HR: 0.56; 95% CI: 0.26–1.21; p = 0.14. rapid vs. slow: HR: 9.32; 95% CI: 3.87–22.5; p < 0.001
			**Visceral metastases** present vs. absent)	Including myeloma: *Univariate*: p = 0.002. *Multivariate*: HR: 2.17; 95% CI: 1.15–4.09; p = 0.017. Excluding myeloma: *Univariate*: p = 0.004. *Multivariate*: HR: 2.42; 95% CI: 1.25–4.64; p = 0.008
			Pathological fracture (present vs. absent)	Including myeloma: *Univariate*: p = 0.929. *Multivariate*: HR: 1.3; 95% CI: 0.68–2.49; p = 0.426. Excluding myeloma: *Univariate*: p = 0.131. *Multivariate*: HR: 1.58; 95% CI: 0.8–3.09; p = 0.182
			Number of spinal metastases (> 1 vs. 1)	Including myeloma: *Univariate*: p = 0.311. *Multivariate*: HR: 1.37; 95% CI: 0.52–3.63; p = 0.521. Excluding myeloma: *Univariate*: p = 0.923. *Multivariate*: HR: 0.49; 95% CI: 0.17–1.4; p = 0.185
			Number of extraspinal bone metastases (≥ 1 vs. none)	Including myeloma: *Univariate*: p = 0.774. *Multivariate*: HR: 0.95; 95% CI: 0.32–2.73; p = 0.916. Excluding myeloma: *Univariate*: p = 0.457. *Multivariate*: HR: 3.16; 95% CI: 0.96–10.4; p = 0.058
			Karnofsky score (low vs. intermediate vs. high)	Including myeloma: *Univariate*: p = 0.027. *Multivariate*: overall: p = 0.08. intermediate vs. high: HR: 1.8; 95% CI: 0.49–6.55; p = 0.375. low vs. high: HR: 3.24; 95% CI: 0.89–11.8; p = 0.075. Excluding myeloma: *Univariate*: p < 0.001. *Multivariate*: overall: p = 0.096. intermediate vs. high: HR: 1.35; 95% CI: 0.35–5.15; p = 0.665. low vs. high: HR: 2.54; 95% CI: 0.68–9.43; p = 0.163
			Neurologic symptoms (MRC 0-3/5 vs. 4-5/5)	Including myeloma: *Univariate*: p = 0.922. *Multivariate*: HR: 1.33; 95% CI: 0.69–2.54; p = 0.386. Excluding myeloma: *Univariate*: p = 0.982. *Multivariate*: HR: 1.02; 95% CI: 0.51–2.02; p = 0.952
			Spinal localisation (cervical vs. thoracic vs. lumbar)	Including myeloma: Univariate: p = 0.638. Excluding myeloma: Univariate: p = 0.371
			Frankel grade (A, B vs. C,D vs. E)	Including myeloma: Univariate: p = 0.930. Excluding myeloma: Univariate: p = 0.976
Liang, 2013, Retrospective (III)	Single center February 2000 to September 2010 n = 92 patients ≥ 60 years old All primaries, including one lymphoma Mean age: 68 39 F: 53 M	Univariate: Kaplan-Meier and Log-rank test Multivariate: Variables with p < 0.05 in univariate analyses were included in the multivariate Cox PH analysis	**Original Tokuhashi score** (1–4 points vs. 5–8 points vs. 9–12 points)	*Univariate*: p = 0.000. *Multivariate*: HR: 0.273; 95% CI: 0.164–0.454; p = 0.000
			**Primary tumor Tomita grade** (rapid vs. moderate vs. slow)	*Univariate*: p = 0.001. *Multivariate*: HR: 2.039; 95% CI: 1.361–3.055; p = 0.001
			Revised Tokuhashi score (1–8 vs. 9–11 vs. 12–15 points)	*Univariate*: p = 0.000. *Multivariate*: non-significant at p < 0.05 but result NR
			Tomita stage (intravertebral vs. perivertebral vs. adjacent vertebral vs. multiple vertebral involvement)	*Univariate*: p = 0.018. *Multivariate*: non-significant at p < 0.05 but result NR
			Tomita score (2–3 vs. 4–5 vs. 6–7 vs. 8–10 points)	*Univariate*: p = 0.000. *Multivariate*: non-significant at p < 0.05 but result NR
			Frankel score (A, B vs. C vs. D, E)	*Univariate*: p = 0.008. *Multivariate*: non-significant at p < 0.05 but result NR
			VAS score (1–4 vs. 5–7 vs. 8–10 points)	*Univariate*: p = 0.018. *Multivariate*: non-significant at p < 0.05 but result NR
			Extraspinal bone involved	*Univariate*: p = 0.038. *Multivariate*: non-significant at p < 0.05 but result NR
			Age (≥60 to <70 vs. ≥70 years)	Univariate: p = 0.468
			KPS (0–40% vs. 50–70% vs. 80–100%)	Univariate: p = 0.686
			Visceral metastasis	Univariate: p = 0.827
			Primary surgery	Univariate: p = 0.062
			Pathological fracture	Univariate: p = 0.056
			Surgical complications	Univariate: p = 0.283
Mollahoseini, 2011, Prospective (III)	Single center February 2007 to March 2009 n = 109 All primaries, (primaries included in “others” are not specified, thus inclusion of hematologic primaries is unclear) Mean age: 57 58 F: 53 M	Univariate: NR Multivariate:Cox PH model 2 Cox PH models were created, which included: (1) Age, sex and Tokuhashi revised score; (2) the 6 variables of the Tokuhashi revised score	(1) Age (continuous), Sex (female vs. male). **Tokuhashi score** (continuous)	Multivariate: only Tokuhashi revised score: -2 log-likelihood = 376.051, χ^2^ = 57.48, df = 1; p < .001. Age and sex: reported to be non-significant at p < 0.05 but result NR
			(2) Cox PH model including the 6 variables comprised in the Tokuhashi revised score: -2 log-likelihood = 380.788, χ^2^ = 71.313, df = 5; p < 0.001	
			**KPS** (10–40% vs. 50–70% vs. 80–100%)	Multivariate: HR: 0.54; 95% CI: 0.32–0.92; p = 0.022
			**Spinal metastases** (1 vs. 2 vs. ≥ 3)	Multivariate: HR: 0.4; 95% CI: 0.25–0.65; p < 0.001
			**Metastases to major internal organs** (unremovable vs. removable vs. no metastasis)	Multivariate: HR: 0.64; 95% CI: 0.48–0.85; p = 0.002
			**Site of primary cancer** (lung, osteosarcoma, stomach, bladder, esophagus, pancreas vs. liver, gallbladder, unidentified vs. others vs. kidney, uterus vs. rectum vs. thyroid, breast, prostate, carcinoid)	Multivariate: HR: 0.62; 95% CI: 0.54–0.72; p < 0.001
			Frankel score (A, B vs. C, D vs. E)	Multivariate: HR: 0.65; 95% CI: 0.42–1; p = 0.055*
			Extraspinal bone metastases (0 vs. 1–2 vs. ≥ 3)	Multivariate: p = 0.686 (HR and 95% CI are NR)
Nemelc, 2014, Retrospective (III)	Single center 2000 to 2010 n = 81 All primaries including myeloma Median age: 59 37 F: 44 M	Cox PH model for both uni- and multivariate Variables with p < 0.20 in univariate analyses were included in the multivariate analysis	**Site of primary cancer** (breast, renal, myeloma, lung, prostate, colorectal, other)	*Univariate*: HR: NR; 95% CI: NR; p = 0.01. *Multivariate*: overall: p < 0.05. renal vs. breast: HR: 2.02; 95% CI: 0.95–4.30; p-value: NR. myeloma vs. breast: HR: 0.22; 95% CI: 0.05–0.95; p-value: NR. lung vs. breast: HR: 4.52; 95% CI: 1.73–11.78; p-value: NR. prostate vs. breast: HR: 1.54; 95% CI: 0.61–3.86; p-value: NR. colorectal vs. breast: HR: 5.53; 95% CI: 2.23–13.74; p-value: NR. others vs. breast: HR: 2.53; 95% CI: 1.6–5.53; p-value: NR
			**ASIA score** (B vs. C vs. D vs. E)	*Univariate*: HR: NR; 95% CI: NR; p = 0.01. *Multivariate*: overall: p < 0.05. ASIA D vs. E: HR: 1.75; 95% CI: 0.63–4.81; p-value: NR. ASIA C vs. E: HR: 13.03; 95% CI: 0.97–3.20; p-value: NR, but in the text: *ASIA C vs*. *E*: *95% CI*: *4*.*57–37*.*16*. ASIA B vs. E: HR: 1.75; 95% CI: 0.63–4.81; p-value: NR
			Visceral metastases (present vs. absent)	*Univariate*: HR: NR; 95% CI: NR; p = 0.18. *Multivariate*: HR: NR; 95% CI: NR; p = 0.57
			Multiple metastases	Univariate: HR: NR; 95% CI: NR; p = 0.82
			Cervical location (yes vs. no)	Univariate: HR: NR; 95% CI: NR; p = 0.89
			Thoracic location (yes vs. no)	Univariate: HR: NR; 95% CI: NR; p = 0.79
			Lumbar location (yes vs. no)	Univariate: HR: NR; 95% CI: NR; p = 0.65
			Sacral location (yes vs. no)	Univariate: HR: NR; 95% CI: NR; p = 0.95
			Radiotherapy (received vs. no)	Univariate: HR: NR; 95% CI: NR; p = 0.33
			Surgical indication (pain vs. instability vs. neurologic impairment vs. neurologic impairment with pain vs. pain with instability vs. neurologic impairment with instability)	Univariate: HR: NR; 95% CI: NR; p = 0.56
Tomita, 2001, Retrospective (III)	Single center 1987 –end of 1992 n = 57 All primaries, no hematologic cancer included Mean age: 56.3 36 F: 31 M	Multivariate: Cox PH analysis	**Primary tumor Tomita grade** (slow vs. moderate vs. rapid)	Multivariate: overall: p < 0.05. moderate vs. slow: HR: 1.82; 95% CI: NR; p-value: NR. rapid vs. slow: HR: 4.08; 95% CI: NR; p-value: NR
			**Visceral metastasis to vital organs** (no vs. treatable by operation or trans-arterial embolisation vs. untreatable)	Multivariate: overall: p < 0.05. treatable vs. no: HR: 1.00; 95% CI: NR; p-value: NR. untreatable vs. no: HR: 1.90; 95% CI: NR; p-value: NR
			**Bone metastases,** spinal metastasis included (solitary/isolated spinal with any other bone metastasis vs. multiple spinal metastasis ± other bone metastasis)	Multivariate: HR: 1.94; 95% CI: NR; p < 0.05
Williams, 2009, Retrospective (III)	Single center 1993–2005 n = 44 Prostate cancer Median age: 68 0 F: 44 M	Cox PH model for both uni- and multivariate Variables with p < 0.15 in univariate analyses were included in multivariate analysis and tested through a backward stepwise selection	**Gleason score** (< 8, median score = 8)	*Univariate*: NR. *Multivariate*: HR: NR; 95% CI: NR; p = 0.002
			**Total number metastases** (≤ 5, median = 5)	*Univariate*: NR. *Multivariate*: HR: NR; 95% CI: NR; p = 0.001
			**Lymph node metastases at time of spinal surgery** (absence vs. presence)	*Univariate*: NR. *Multivariate*: HR: NR; 95% CI: NR; p = 0.04
			**Degree compression of spinal canal** (≤ 25%)	*Univariate*: NR. *Multivariate*: HR: NR; 95% CI: NR; p = 0.001
Chen, 2015, Retrospective (III)	Single centre November 2000 to March 2010 n = 50 NSCLC Mean age: 61.6 16 F: 34 M	Cox PH model for both uni- and multivariate Variables with p < 0.05 in univariate analyses were included in multivariate analysis Although palsy score was not significant on univariate analysis, it was considered to be an important factor, so it was included in multivariate analysis	**KPS** (10–40% vs. 50–70% vs. 80–100%)	*Univariate*: 50–70 vs. 10–40%: HR: 0.43; 95% CI: 0.18–1.03; p = 0.059. 80–100 vs. 10–40%: HR: 0.09; 95% CI: 0.03–0.26; p<0.001. *Multivariate*: 50–70 vs. 10–40%: HR: 0.52; 95% CI: 0.16–1.74; p = 0.289. 80–100 vs. 10–40%: HR: 0.14; 95% CI: 0.03–0.54; p = 0.004
			Age (≤54 vs. 55–74 vs. ≥75)	*Univariate*: 55–74 vs. ≤54: HR: 1.16; 95% CI: 0.60–2.25; p = 0.659. ≥75 vs. ≤54: HR: 3.28; 95% CI: 1.37–7.82; p = 0.008. *Multivariate*: 55–74 vs. ≤54: HR: 0.78; 95% CI: 0.37–1.64; p = 0.512. ≥75 vs. ≤54: HR: 1.22; 95% CI: 0.37–4.05; p = 0.748
			Frankel score (A, B vs. C, D)	*Univariate*: HR: 1.18; 95% CI: 0.49–2.83; p = 0.706. *Multivariate*: HR: 1.23; 95% CI: 0.50–3.03; p = 0.653
			Tumor histology (adenocarcinoma vs. non-adenocarcinoma)	*Univariate*: HR: 0.38; 95% CI: 0.20–0.71; p = 0.003. *Multivariate*: HR: 0.59; 95% CI: 0.28–1.25; p = 0.167
			Sex (female vs. male)	Univariate: HR: 0.61; 95% CI: 0.33–1.14; p = 0.120
			Number of vertebra involved (<3 vs. ≥3)	Univariate: HR: 0.70; 95% CI: 0.39–1.25; p = 0.228
			Other bone metastasis (absent vs. present)	Univariate: HR: 0.83; 95% CI: 0.46–1.49; p = 0.531
			Visceral metastasis (absent vs. present)	Univariate: HR: 1.08; 95% CI: 0.52–2.23; p = 0.837
			BMI (underweight vs. eutrophic vs. overweight/obese)	Univariate: eutrophic vs. underweight: HR: 1.03; 95% CI: 0.39–2.68); p = 0.958. overweight vs. underweight: HR: 0.72; 95% CI: 0.25–2.05); p = 0.538
Lei, 2015 Retrospective (III) *Development of a scoring system*	Single center May 2005 to May 2015 n = 64 NSCLC Median age: 57 22 F: 42 M	Univariate Kaplan-Meier and Log-rank test; Cox PH model Multivariate Variables with p < 0.05 in univariate analyses were included in the Cox PH model following stepwise selection	**ECOG performance status** (1–2 vs. 3–4)	*Univariate*: Log-rang test: p < 0.001. Cox PH model: HR: 2.78; 95% CI: 1.54–5.02; p < 0.001. *Multivariate*: HR: 2.18; 95% CI: 1.15–4.16; p = 0.017
			**Number of vertebrae involved** (1–2 vs. ≥ 3)	*Univariate*: Log-rang test: p < 0.001. Cox PH model: HR: 2.46; 95% CI: 1.39–4.35; p = 0.002. *Multivariate*: HR: 2.05; 95% CI: 1.11–3.76; p = 0.021
			**Visceral metastasis** (absent vs. present)	*Univariate*: Log-rang test: p = 0.002. Cox PH model: HR: 2.29; 95% CI: 1.33–3.94; p = 0.003. *Multivariate*: HR: 2.00; 95% CI: 1.10–3.62; p = 0.022
			**Time developing motor deficits before surgery** (≤ 14 vs. > 14 days)	*Univariate*: Log-rang test: p < 0.001. Cox PH model: HR: 3.44; 95% CI: 1.90–6.22; p, 0.001. *Multivariate*: HR: 2.70; 95% CI: 1.45–5.03; p = 0.002
			Ambulatory status (ambulatory vs. non-ambulatory)	*Univariate*: Log-rang test: p = 0.003. Cox PH model: HR: 2.24; 95% CI: 1.30–3.86; p = 0.004. *Multivariate*: non-significant at p < 0.05 but result NR
			Age (≤ 57 vs. >57, median 57 years)	Univariate: Log-rang test: p = 0.16
			Sex (female vs. male)	Univariate: Log-rang test: p = 0.90
			Other bone metastases (absent vs. present)	Univariate: Log-rang test: p = 0.58
			Interval from cancer diagnosis to surgery (≤ 80 vs. >80 days, median time: 80 days)	Univariate: Log-rang test: p = 0.73
Lei, 2015, Retrospective (III)	Single center May 2005 to May 2015 n = 37 (test group) Lung cancer, including NSCLC (n = 33) and SCLC (n = 4) Median age: 57 12 F: 25 M	Univariate: Kaplan-Meier and Log-rank test Multivariate:Variables with p < 0.05 in univariate analyses were included in the multivariate Cox PH model following a stepwise selection	**Visceral metastasis** (absent vs. present)	*Univariate*: p = 0.0118. *Multivariate*: Risk ratio: 7.913; 95% CI: 2.678–23.382; p = 0.0002
			**Time developing motor deficits before surgery** (≤14 vs. >14 days)	*Univariate*: p < 0.0001. *Multivariate*: Risk ratio: 4.828; 95% CI: 2.005–11.628; p = 0.0004
			**Ambulatory status** (ambulatory vs. non-ambulatory)	*Univariate*: p = 0.0054. *Multivariate*: Risk ratio: 4.510; 95% CI: 1.757–11.578; p = 0.0017
			Number of vertebrae involved (1–2 vs. ≥ 3)	*Univariate*: p = 0.0028. *Multivariate*: non-significant at p < 0.05 but result NR
			ECOG performance status (1–2 vs. 3–4)	*Univariate*: p = 0.0002. *Multivariate*: non-significant at p < 0.05 but result NR
			Age (≤ 57 vs. >57, median 57 years)	Univariate: p = 0.3802
			Sex (female vs. male)	Univariate: p = 0.5626
			Histology (adenocarcinoma vs. non-adenocarcinoma	Univariate: p = 0.2288
			Other bone metastases (absent vs. present)	Univariate: p = 0.8718
			Interval from cancer diagnosis to surgery (≤ 80 vs. >80 days, median time: 80 days)	Univariate: p = 0.6304

Bolted predictive factors are statistically significant in multivariate analysis at a significance level of p < 0.05

^†^ Study sample characteristics: Number of center(s) involved; Study span; Patients (n); Primary tumor included; Age at surgery; Female:Male (F:M)

^‡^ For predictive factors used as categorical variables, the referent is underlined only when it was clearly reported in a table or specified in the text.

* The authors report Frankel score as being a statistically significant predictor when they reported setting p < 0.05 as significant.

Abbreviations (alphabetical order): **95% CI**: 95% confidence interval; **ASA**: American society of anesthesiologists physical status classification; **ASIA**: American spinal injury association; **BMI**: body mass index; **ECOG**: Eastern Cooperative Oncology Group; **GTR**: gross-total resection; **HR**: hazard ratio; **KPS**: Karnofsky performance status; **mo**: months; **MRC**: medical research council motor strength scale **MS**: median survival; **NR**: not reported; **NSCLC**: non-small cell lung cancer; **OR**: odd ratio; **PH**: proportional hazard; **PHA**: proportional hazard assumption; **PSA**: prostate-specific antigen; **SCLC**: small cell lung cancer; **SSM**: symptomatic spinal metastasis; **STR**: sub-total resection; **ukn**: unknown; **vs.**: versus

Prognostic factors for survival varied substantially according to primary tumor types, with negative relationship as follows: breast cancer with shorter time interval from cancer diagnosis to SSM surgery, emergency hospital admission, primary tumor with poor/undifferentiated histologic grade and negative progesterone receptors[48]; HHC with low serum albumin and high lactate dehydrogenase [52]; prostate cancer with KPS 50–70%[54], Gleason score > 8[49], total number of metastases > 5[49], presence of lymph node metastases[49], and degree of canal compression > 25%[49]; lung cancer with presence of visceral metastasis, ≤ 14 days from onset of motor deficit to surgery, incapacity to walk independently or with a walking aid [55]; NSCLC with KPS < 80%[51], ECOG 3–4[41], ≥ 3 vertebral metastases[41], presence of visceral metastasis[41] and ≤ 14 days from onset of motor deficit to surgery[41]; and unknown site of primary tumor with cervical spinal location, Frankel score A, B or C, and presence of extraspinal disease at presentation[47].

### Methodological issues

All 17 studies used the Cox proportional hazards (PH) regression method for their multivariate survival analysis. Five studies[24, 43, 46, 47, 49] did not provide a clear definition, measurement or categorization of their predictors, e.g. “High versus Intermediate versus Low KPS” without defining the corresponding KPS numerical range. One study[50] identified CCIS ≥ 2 as a predictor of survival although CCIS ≥ 2 is not a discriminatory factor given that the sole presence of metastatic solid tumor gives a CCIS of 6[58]. Four studies[43, 44, 49, 53] did not clearly report which predictors were assessed using univariate analysis and, among these studies, one[49] did not report any results for these analyses. Three studies[52, 53, 57] did not specify how predictors were selected to enter the multivariate analysis. Four studies[24, 48, 50, 51] did not analyze predictors that were described in the Methods section, and two studies[47, 53] did not clearly distinguish the results from uni- and multivariate analyses. Only three[43, 48, 50] studies mentioned testing for the proportional hazards assumption (PHA). While two[48, 50] of these studies specified the statistical method used to test the PHA, none actually reported their result. One study[43] reported testing for collinearity but reported neither the technique used nor the results. Eight studies [45, 51, 55, 56],[43, 48, 49, 53] did not report how many patients died during follow-up. Among these, four[45, 51, 55, 56] included more predictor degrees of freedom in their multivariate model than their total sample size *n* divided by 10. In addition, six studies[44, 51, 53–56] included at least one predictor that had ten or less events in a stratum in one of their categorical variable(s). Furthermore, deficiencies in reporting were identified in all 17 studies, including: three studies[47, 49, 53] did not report the hazard ratio or confidence intervals, seven studies[43, 46, 48, 49, 52, 55, 56] studies did not identify the referent stratum for their categorical predictors and 12 studies[24, 41, 44–46, 49, 50, 53–57] did not indicate the directionality of associations (shorter or longer survival).

### Overall strength of evidence related to survival

Seven studies examined the preoperative clinical factors associated with survival in patients with SSM from all sites of primary tumor including multiple myeloma (MM). A total of 20 factors were identified, among which 11 were related to the site/histology of the primary tumor[24, 43–45, 50, 56, 57]. Two studies[24, 59] evaluated predictors of survival in patients with SSM from all sites of primary tumors excluding MM and reported three predictors: primary tumor Tomita Grade II and III (original classification), presence of visceral metastases and presence of bone metastases (spinal and extraspinal). The baseline strength of evidence was *Low* for preoperative prognostic factors of survival in studies that considered all sites of primary tumors including or excluding MM. When MM was included, only radioresistant site of primary tumor had a final strength of evidence of *Low*. All other predictors related to the site of primary tumor were downgraded to *Very low* due to high risk of bias related to inconsistency of results. Non-ambulatory status and CCIS ≥ 2 maintained a final strength of evidence of *Low* while the remaining seven predictors were downgraded to *Very low* for at least high risk of bias. The final strength of evidence for the three predictors of survival for all primaries excluding MM was also *Very low* for at least high risk of bias (Table 3).

**Table 3:** Overall body of evidence for preoperative predictors of survival in surgical SSM patients from multiple studies.

Negative preoperative predictors	Baseline strength evidence	Univariate consistent	Univariate inconsistent	Multivariate consistent	Multivariate inconsistent	Up- / Downgrade	Final strength evidence
**All types of primary tumors, including multiple myeloma**[24, 43–45, 50, 56, 57]							
Modified primary tumor Tomita Grade III, i.e. rapid growing tumor*	Low	Bollen, 2013: *rapid vs*. *moderate vs*. *slow*		Bollen, 2013: *rapid vs*. *slow*	Bollen, 2013: *mod*. *vs*. *slow*	-1: risk of bias	Very low
Primary tumor Tomita Grade III, i.e. rapid growing tumor**	Low	Leithner, 2008: *rapid vs*. *moderate vs*. *slow*		Leithner, 2008: s*rapid vs*. *slow*	Leithner, 2008: *mod*. *vs*. *slow*	-1: risk of bias	Very low
Poor prognosis primary tumor***	Low	Hosono, 2005: *favorable (myeloma*, *renal*, *thyroid*, *breast*, *prostate) vs*. *poor prognosis (other primaries)*		Hosono, 2005: *favorable (myeloma*, *renal*, *thyroid*, *breast*, *prostate) vs*. *poor prognosis (other primaries)*		-1: risk of bias	Very low
Radioresistant primary tumor****	Low	Arrigo, 2011: *radioresistant vs*. *radiosensitive*		Arrigo, 2011: *radioresistant vs*. *radiosensitive*			Low
Lung primary	Low		Arrigo, 2011: *lung vs*. *breast vs*. *prostate vs*. *renal vs*. *colon vs*. *other*	Finkelstein, 2003: *lung vs*. *other*		-1: risk of bias -1: consistency	Very low
Melanoma primary	Low			Finkelstein, 2003 *melanoma vs*. *other*		-1: risk of bias	Very low
Breast primary	Low		Arrigo, 2011: *lung vs*. *breast vs*. *prostate vs*. *renal vs*. *colon vs*. *other*	Finkelstein, 2003 *breast vs*. *other*		-1: risk of bias -1: consistency	Very low
Stomach primary	Low			Finkelstein, 2003: *stomach vs*. *other*		-1: risk of bias	Very low
Colon primary	Low		Arrigo, 2011: *lung vs*. *breast vs*. *prostate vs*. *renal vs*. *colon vs*. *other*	Finkelstein, 2003: *colon vs*. *other*		-1: risk of bias -1: consistency	Very low
Prostate primary	Low		Arrigo, 2011: *lung vs*. *breast vs*. *prostate vs*. *renal vs*. *colon vs*. *other*	Finkelstein, 2003: *prostate vs*. *other*		-1: risk of bias -1: consistency	Very low
Kidney primary	Low		Arrigo, 2011: *lung vs*. *breast vs*. *prostate vs*. *renal vs*. *colon vs*. *other*	Finkelstein, 2003: *kidney vs*. *other*		-1: risk of bias -1: consistency	Very low
Non-ambulatory	Low	Arrigo, 2011: *ambulatory ± walking aid vs*. *not ambulatory*		Arrigo, 2011: *ambulatory ± walking aid vs*. *not ambulatory*			Low
Presence of neurologic deficit or palsy or Frankel/ASIA other than E	Low	Hosono, 2005: *paretic vs*. *non-paretic* Nemelc, 2014: *ASIA B vs*. *C vs*. *D vs*. *E*	Arrigo, 2011: *Frankel E vs*. *C*,*D vs*. *A*,*B* Bollen, 2013: *Frankel E vs*. *C*,*D vs*. *A*,*B* Leithner, 2008: *MRC 0-5/5 vs*. *4-5/5* Leithner, 2008: *Frankel E vs*. *C*,*D vs*. *A*,*B*	Finkelstein, 2003: *present vs*. *absent* Hosono, 2005: *paretic vs*. *non-paretic* Nemelc, 2014: *ASIA C vs*. *E*	Leithner, 2008: *MRC 0-5/5 vs*. *4-5/5* Mollahoseini, 2011: *Frankel E vs*. *C*,*D vs*. *A*,*B* Nemelc, 2014: *ASIA D vs*. *E; ASIA B vs*. *E*	-1: risk of bias -1: consistency	Very low
Charlson comorbidity index score ≥ 2	Low	Arrigo, 2011: *0–1 vs*. *≥ 2*		Arrigo, 2011: *0–1 vs*. *≥ 2*			Low
KPS 10–40%	Low	Bollen, 2013: *rapid vs*. *moderate vs*. *slow*	Arrigo, 2011: *independent vs*. *partially/fully independent functional status* Leithner, 2008: *low vs*. *intermediate vs*. *high*	Bollen, 2013: *10–40 vs*. *80–100%* Mollahoseini, 2011; *10–40 vs*. *50–70 vs*. *80–100%*	Bollen, 2013: *50–70 vs*. *80–100%* Leithner, 2008: *Intermediate vs*. *high; intermediate vs*. *low; p = 0*.*075*	-1: risk of bias -1: consistency	Very low
Presence of visceral metastases	Low	Bollen, 2013: *present vs*. *absent* Leithner, 2008; *present vs*. *absent*	Arrigo, 2011: *present vs*. *absent* Bollen, 2013; *treatable vs*. *non-treatable vs*. *absent* Nemelc, 2014; *present vs*. *absent*	Bollen, 2013; *present vs*. *absent* Leithner, 2008; *present vs*. *absent* Mollahoseini, 2011; *treatable vs*. *non-treatable vs*. *absent*	Arrigo, 2011; *present vs*. *absent* Nemelc, 2014; *present vs*. *absent*	-1: risk of bias -1: consistency	Very low
Number of spinal metastasis	Low		Arrigo, 2011; *continuous variable* Bollen, 2013; *1 vs*. *2 vs*. *≥3* Leithner, 2008; *1 vs*. *>1*	Mollahoseini, 2011; *1 vs*. *2 vs*. *≥ 3*	Leithner, 2008; *1 vs*. *>1*	-1: risk of bias -1: consistency	Very low
Older age	Low		Arrigo, 2011; *continuous variable* Bollen, 2013; *<65 vs*. *≥ 65 years*	Finkelstein, 2003; *continuous variable*	Arrigo, 2011; *continuous variable* Mollahoseini, 2011; *continuous variable*	-1: risk of bias -1: consistency	Very low
Male sex	Low		Arrigo, 2011 *male vs*. *female* Bollen, 2013 *male vs*. *female*	Finkelstein, 2003 *male vs*. *female*	Mollahoseini, 2011 *male vs*. *female*	-1: risk of bias -1: consistency	Very low
Presence of pain	Low	Hosono, 2005; *pain vs*. *no pain*		Hosono, 2005; *pain vs*. *no pain*		-1: risk of bias	Very low
**All types of primary tumors, excluding multiple myeloma**[24,53]							
Primary tumor Tomita Grade II and III, i.e. moderate and rapid growing tumor, respectively	Low	Leithner, 2008		Tomita, 2001: *mod*. *vs*. *slow; rapid vs*. *slow* Leithner, 2008: *rapid vs*. *slow*	Leithner, 2008: *mod*. *vs*. *slow*	-1: risk of bias	Very low
Presence of visceral metastases	Low	Leithner, 2008: *present vs*. *absent*		Tomita, 2001: *treatable vs*. *non-treatable vs*. *absent* Leithner, 2008: *present vs*. *absent*		-1: risk of bias	Very low
Presence of bone metastases, both spinal and extraspinal	Low		Leithner, 2008: (a) number of spinal metastases *(>1 vs*. *1*); (b) number of extraspinal bone metastases (*≥ 1 vs*. *1*)	Tomita, 2001: *solitary spinal ± extra-spinal vs*. *multiple spinal ± extra-spinal metastasis*	Leithner, 2008: (a) number of spinal metastases *(>1 vs*. *1*); (b) number of extraspinal bone metastases *(≥ 1 vs*. *1*); p = 0.058	-1: risk of bias -1: consistency	Very low
**Prostate cancer**[49,54]							
50–70% KPS	Low					-1: risk of bias	Very low
Gleason score > 8	Low					-1: risk of bias	Very low
Total number metastases > 5	Low					-1: risk of bias	Very low
Presence of metastases to lymph node at the time of spinal surgery	Low					-1: risk of bias	Very low
> 25% spinal canal compression	Low					-1: risk of bias	Very low
**NSCLC**[41, 51]							
Low performance status	Low	Chen, 2015: KPS: 80–100 vs.10–40% Lei, 2015: ECOG: *1–2 vs*. *3–4*	Chen, 2015: KPS: 50–70 vs. 10–40%; p = 0.059	Chen, 2015: KPS: 80–100 vs.10–40% Lei, 2015: ECOG: *1–2 vs*. *3-4s*	Chen, 2015: KPS: 50–70 vs. 10–40%	-1: risk of bias	Very low
≥ 3vertebrae involved	Low	Lei, 2015: *1–2 vs*. *≥ 3*	Chen, 2015	Lei, 2015: *1–2 vs*. *≥ 3*		-1: risk of bias -1: consistency	Very low
Present of visceral metastasis	Low	Lei, 2015	Chen, 2015	Lei, 2015		-1: risk of bias -1: consistency	Very low
≤ 14 days between development of motor deficits to surgery	Low					-1: risk of bias	Very low

* Mollahoseini, 2011 identified the following “Site of primary cancer”: lung, osteosarcoma, stomach, bladder, esophagus, pancreas vs. liver, gallbladder, unidentified vs. others vs. kidney, uterus vs. rectum vs. thyroid, breast, prostate, carcinoid

** Nemelc, 2014 identified the following “Site of primary cancer”: breast, renal, myeloma, lung, prostate, colorectal, other

*** Hosono, 2005 defined “primary tumor with favorable prognosis” as those with more than 20 months median survival, which included myeloma, thyroid, kidney, breast, and prostate; and primary tumor with poor prognosis as those with less than 20 months median survival (lung, sarcoma, liver, colon, stomach, uterus, head and neck, bladder, thymus, pancreas, esophagus, and unknown)

**** Arrigo, 2011 defined “radiosensitive tumors” as breast, prostate, hematogenous, small cell lung, and germ cell; and all other tumors were classified as “radioresistant”

Abbreviations (alphabetical order): **ASIA**: American spinal injury association; **ECOG**: Eastern Cooperative Oncology Group; **KPS**: Karnofsky performance status; **NSCLC**: non-small cell lung cancer; **SSM**: symptomatic spinal metastasis; **vs.**: versus

Various preoperative factors of survival were identified in multivariate analysis in specific groups of SSM patients. Although two studies examined the preoperative factors of survival in patients with SSM from prostate cancer, they did not consider the same predictors. KPS 50–70%[54], Gleason score > 8[49], total number of metastases > 5[49], presence of lymph node metastases at the time of surgery[49] and degree of canal compression > 25%[49] had a *Low* strength of evidence at baseline and their respective final strength of evidence was *Very low* due to high risk of bias (Table 3). Based on two studies[41, 51], the four predictors of survival for SSM resulting from NSCLC also had a *Low* strength of evidence at baseline. All predictors were downgraded to a *Very low* final strength of evidence: low performance status and ≤ 14 days from onset of motor deficit to surgery because of high risk of bias and ≥ 3 vertebral metastases and presence of visceral metastasis because of high risk of bias and inconsistency (Table 3). Preoperative prognostic factors in patients with (1) unknown site of primary tumor at the time of SSM surgery, (2) breast cancer, (3) HHC, (4) lung cancer, ≥ 60 years old with SSM from heterogenous primary tumors were derived from a single study, all of which had a *Low* strength of evidence at baseline. The final strength of evidence for predictors of survival in breast cancer[48] was *Low* while all the others[46, 47, 52, 55] were downgraded to *Very low* due to high risk of bias[46, 47, 52, 55] and imprecision[47] (Table 4).

**Table 4:** Overall body of evidence for preoperative predictors of survival in surgical SSM patients from a single study.

Negative preoperative predictors	Baseline strength	Up- / Downgrade	Final strength of evidence
**Breast cancer** [48]			
Longer time interval from cancer diagnosis to surgery (year)	Low		Low
Admission to hospital via emergency room			
Poor/undifferentiated histologic grade			
Negative progesterone receptors			
**Hepatocellular carcinoma**[52]			
Serum albumin <37 g/L	Low	-1: risk of bias	Very low
Lactate dehydrogenase ≥ 200 U/L			
**Lung cancer: NSCLC and SCLC**[55]			
Presence of visceral metastasis	Low	-1: risk of bias	Very low
≤14 days between development of motor deficits due to SSM to surgery			
Non-ambulatory status			
**≥ 60 years old patients**[46]			
Original Tokuhashi score < 9 points	Low	-1: risk of bias	Very low
Rapid- or moderate-growing primary tumour Tomita grade			
**Unknown site of primary tumor at the time of SSM surgery**[47]			
Cervical spinal location	Low	-1: risk of bias -1: precision	Very low
Presence of extraspinal disease at presentation			
Frankel A, B, C			

Abbreviations (alphabetical order): **NSCLC**: non-small cell lung cancer; **SCLC**: small cell lung cancer; **SSM**: symptomatic spinal metastasis

## Discussion

### Summary of findings

To our knowledge, this is the first systematic literature review that has sought to determine the key preoperative predictors of survival (KQ1), neurologic (KQ2), functional (KQ3), and HRQoL (KQ4) outcomes in patients with SSM who underwent surgical treatment. This systematic review identified 17 studies related to our KQ1 that conducted multivariate analysis and reported a total of 46 preoperative prognostic factors of survival in surgical SSM patients. All 17 prognostic studies were rated as having a moderately high risk of bias (Class III evidence). The final strength of the overall body of evidence was graded *low* for 7 and *very low* for the remaining 39 predictors of survival.

In spite of performing a literature search designed to maximize sensitivity, this review was only able to identify studies addressing KQ1. Six studies examined the clinical prognostic factors of functional status (KQ3) and one study isolated predictors of HRQoL (KQ4), but these studies were excluded because they included surgical and/or postoperative predictors. Inclusion of such predictors in the multivariate analysis runs the risk of precluding relevant preoperative predictors from either being selected in the final model or showing statistical significance. Also, final models that retain surgical/postoperative predictors are not relevant in the preoperative period, which is the critical time-point for clinical decision-making. Therefore, while this review had limited success in establishing preoperative prognostic factors of survival, it also highlights the dearth of evidence related to predictors of neurologic, functional, and HRQoL outcomes in surgical SSM patients.

### Methodological considerations and recommendations

Due to the nature of cohort studies, prognostic data from these will be biased and may not be generalizable to patients with spinal metastases. Cohort studies are often limited by cost and timescales, and significant losses to follow-up. Spinal centers may cover large geographical areas, and patients may be transferred elsewhere for subsequent oncological treatments. Failure to return for spinal clinic follow-up at prearranged appointment times may be due to travel constraints, the patient may be too unwell, undergoing other treatments, or they may prefer to be reviewed by local oncologists instead.

In addition, survival analyses are inherently complex, and our attempt to synthesize this group of 17 such studies was challenging due to considerable heterogeneity in patient samples and prognostic factors investigated. Furthermore, the design and reporting of the statistical analyses were problematic in many studies, leading to a moderately high risk of bias and difficulty interpreting the results. Since multivariate techniques applied to systematically collected data from a specific patient population may improve clinical prediction by identifying key prognostic factors[23, 60] and it is likely that various factors conjointly influence clinical outcomes such as survival or HRQoL, performing multivariate analysis was one of our inclusion criteria. Conducting multivariate analysis not only helps control for confounders, thus enhancing the confidence in the validity of the study results[61], but also provides an estimate of the actual effect size, offering both a clinical and statistical assessment of the impact of each factor on the outcome variable.[62]

Prognostic studies should be designed and conducted to minimize potential biases related to six domains.[35] (1) Study participants and sample: Data should be collected prospectively. Patients should be at a common point in the course of their disease. Patient sample assembly should include method, period, place of recruitment, and eligibility criteria. Patient sample should be adequately described for key characteristics. (2) Study attrition: The follow-up period should be long enough for the outcome(s) of interest to occur. The proportion of participants completing the study should be reported and adequate for the study design and analyses. If applicable, the reason(s) for loss to follow-up should be recorded. Account and measurement of (3) prognostic factors, (4) outcomes and (5) confounding factors: the definition and method of measurement of prognostic factors, outcomes and confounders should be clearly described, valid, reliable and appropriate. An adequate proportion of the study sample should have complete data for prognostic factor, outcome and confounders, and if imputation is used for missing data, the method should be described and appropriate. (6) Analysis: The statistical analyses, including model selection and building, should be suitable for the study design, assumptions should be verified, and if applicable, adequate adjustment for confounding should be undertaken. Finally, all results should be adequately reported.[35, 63, 64]

### The development of clinical prediction rules

While there is no well recognized CPR for HRQoL, the variable prognostic ability of current CPRs of survival[24–26] in this patient population may be related to the fact that patients who are deemed surgical candidates are fundamentally different, with overall greater life expectancy and fewer comorbidities, than patients selected for conservative or radiotherapy treatment alone. Selecting relevant predictors from a larger set of candidate predictors is one of the steps involved in the first phase of development of CPRs; these predictors are typically derived from best literature evidence. These CPRs could be of high clinical value by providing more accurate estimates of survival and HRQoL after surgery, helping not only to guide therapeutic decision-making during informed consent discussions, but also patients to form more realistic expectations relative to surgical outcomes.

### Strengths and limitations

The systematic literature review was conducted in accordance with the PRISMA guidelines. We assessed the quality of the studies and evaluated the strength of the overall body of evidence for each preoperative predictor identified through our sensitive and rigorous literature review. However, this review aimed to identify predictors of a wide range of outcomes, combining the results of studies with substantial heterogeneity in the prognostic factors, outcome measures, and patient populations that were assessed, which may constitute a problem with internal validity. Furthermore, our *a priori* eligibility criteria were relatively narrow in their requirement and may have excluded studies that produced pertinent findings.

In predicting future outcomes by using patient data available at presentation, there will always be a degree of randomness or “chaos” in the system affecting clinical outcomes and survival.[65] Although we may improve prediction by establishing better methodology, there will always be random variability between studies and between patients, and there comes a point where studying preoperative patient variables too closely may not be helpful, due to the inherent variation that does not improve regardless of increasing sample size.

### Conclusions

Life expectancy and HRQoL are cornerstones to clinical decision-making in surgical SSM patients. Based on the results of 17 pertinent studies, this systematic review found a low overall strength of evidence for seven preoperative predictors of survival and very low strength evidence for 39 additional predictors. Consequently, we have low confidence that the evidence reflects the true effect size of these predictors. Furthermore, no evidence was found for the prediction of neurologic, functional, and HRQoL outcomes. Further rigorously conducted prospective studies are needed to better understand what preoperative factors are prognostic of these various outcomes, for the purpose of surgical decision-making, development of CPRs, patient education and levering treatment expectations. Genetic analysis of tumor subtypes will also need to be included in future prediction models, since novel chemotherapies and immunotherapies are showing promising influence on survival and HRQoL.

## Supporting information

S1 Checklist PRISMA research checklist. (DOC)

S1 Table Complete search strategies from all seven databases. (DOCX)

S2 Table Different levels of evidence for predictive studies. (DOCX)

S3 Table Rating quality of the overall body of evidence. (DOCX)

## Abbreviations

ASIA: American Spinal Injury Association
CCIS: Charlson comorbidity index score
CPRs: clinical prediction rules
ECOG: Eastern Cooperative Oncology Group
EQ-5D: EuroQol 5 dimensions
FIM: functional independence measure
GRADE: Grading of Recommendation Assessment, Development and Evaluation
HCC: hepatocellular carcinoma
HRQoL: health related quality of life
KPS: Karnofsky performance status
MM: multiple myeloma
MRC: Medical Research Council
NSCLC: non-small cell lung carcinoma
ODI: Oswestry Disability Index
PH: proportional hazards
PHA: proportional hazards assumption
PRISMA: Preferred Reporting Items for Systematic Reviews and Meta-Analyses
PROSPERO: International Prospective Register of Systematic Reviews
SF-36: short form health survey
SSM: symptomatic spinal metastasis
