# Supplementary material for: Symptomatic spinal metastasis: A systematic literature review of the preoperative prognostic factors for survival, neurological, functional and quality of life in surgically treated patients and methodological recommendations for prognostic studies
Source: PLoS One. 2017 Feb 22;12(2):e0171507. doi: 10.1371/journal.pone.0171507 (PMC5321441; doi:10.1371/journal.pone.0171507)
Supplement: S1 Table — (DOCX) [file pone.0171507.s002.docx]

**S1 Table**: Complete search strategies from all seven databases

OVID MEDLINE(R) 1946 to April Week 1 2016

| 1 | Spinal Cord Neoplasms/ | 9485 |
| --- | --- | --- |
| 2 | Spinal Neoplasms/ | 11939 |
| 3 | Epidural Neoplasms/ | 500 |
| 4 | Epidural Space/ | 3975 |
| 5 | ((spine? or spinal) adj3 adenocarcin*).mp. | 21 |
| 6 | ((spine? or spinal) adj3 cancer*).mp. | 354 |
| 7 | ((spine? or spinal) adj3 carcino*).mp. | 156 |
| 8 | ((spine? or spinal) adj3 malign*).mp. | 466 |
| 9 | ((spine? or spinal) adj3 metasta*).mp. | 2826 |
| 10 | ((spine? or spinal) adj3 neoplas*).mp. | 21139 |
| 11 | ((spine? or spinal) adj3 lesion*).mp. | 6725 |
| 12 | ((spine? or spinal) adj3 tumo?r*).mp. | 4494 |
| 13 | (epidural adj3 adenocarcin*).mp. | 4 |
| 14 | (epidural adj3 cancer*).mp. | 83 |
| 15 | (epidural adj3 carcin*).mp. | 17 |
| 16 | (epidural adj3 lesion*).mp. | 234 |
| 17 | (epidural adj3 metasta*).mp. | 354 |
| 18 | (epidural adj3 malign*).mp. | 69 |
| 19 | (epidural adj3 neoplas*).mp. | 549 |
| 20 | (epidural adj3 tumo?r*).mp. | 313 |
| 21 | (epidural adj3 lesion*).mp. | 234 |
| 22 | (spinal adj1 cord? adj1 compress*).mp. | 11649 |
| 23 | (compress* adj4 myelopath???).mp. | 581 |
| 24 | conus medullaris syndrome?.mp. | 51 |
| 25 | (compress* adj6 spin?? cord?).mp. | 12726 |
| 26 | (epidural adj6 compress*).mp. | 905 |
| 27 | or/1-26 [Spinal Cord or Epidural Cancer & related terms] | 42503 |
| 28 | exp Neoplasm Metastasis/ | 170626 |
| 29 | sc.fs. [secondary] | 136471 |
| 30 | Micrometast*.mp. | 5543 |
| 31 | Micro-metast*.mp. | 350 |
| 32 | metasta*.mp. | 397257 |
| 33 | secondary.mp. | 510596 |
| 34 | or/28-33 [Metastasis & related terms] | 926115 |
| 35 | 27 and 34 [Spinal Cord Ca + Metastasis] | 10051 |
| 36 | exp Neurosurgical Procedures/ | 165439 |
| 37 | Surgery, Computer-Assisted/ | 11922 |
| 38 | Surgical Procedures, Minimally Invasive/ | 19328 |
| 39 | Surgical Procedures, Minor/ | 1315 |
| 40 | Surgical Procedures, Elective/ | 9802 |
| 41 | Decompression, Surgical/ | 11629 |
| 42 | Neurosurgery/ | 13586 |
| 43 | Orthopedics/ | 18062 |
| 44 | exp Orthopedic Procedures/ | 233503 |
| 45 | exp cementoplasty/ or vertebroplasty/ or kyphoplasty/ | 1808 |
| 46 | Foraminotomy/ | 54 |
| 47 | Microsurgery/ | 23654 |
| 48 | Metastasectomy/ | 390 |
| 49 | Reconstructive Surgical Procedures/ | 38119 |
| 50 | "Prostheses and Implants"/ | 40938 |
| 51 | balloon?.mp. | 89531 |
| 52 | (bone adj1 cement*).mp. | 11139 |
| 53 | cementoplast*.mp. | 134 |
| 54 | (computer* adj2 aid* adj2 surg*).mp. | 330 |
| 55 | (computer* adj2 assist* adj2 surg*).mp. | 12280 |
| 56 | (decompress* adj2 procedur*).mp. | 829 |
| 57 | (decompress* adj2 surg*).mp. | 15063 |
| 58 | (elective* adj3 surg*).mp. | 22742 |
| 59 | (excise or excises or excised or excising).mp. | 44156 |
| 60 | excision*.mp. | 120283 |
| 61 | (fixation adj2 devic*).mp. | 7153 |
| 62 | fixation?.mp. | 167243 |
| 63 | foraminotom*.mp. | 364 |
| 64 | implant?.mp. | 156411 |
| 65 | kyphoplast*.mp. | 1168 |
| 66 | laminectom*.mp. | 11782 |
| 67 | laminotom*.mp. | 473 |
| 68 | metastasectom*.mp. | 1420 |
| 69 | micro-dissect*.mp. | 481 |
| 70 | microdissect*.mp. | 12203 |
| 71 | (minimal* adj2 invasiv*).mp. | 47494 |
| 72 | (minor adj2 surg*).mp. | 4049 |
| 73 | neurosurg*.mp. | 55336 |
| 74 | operat*.mp. | 825837 |
| 75 | osteoplast*.mp. | 1802 |
| 76 | prosthes*.mp. | 228411 |
| 77 | prosthetic*.mp. | 40650 |
| 78 | (reconstruc* adj2 (spine? or spinal*)).mp. | 570 |
| 79 | (reconstruc* adj2 surg*).mp. | 52290 |
| 80 | remov*3.mp. | 451813 |
| 81 | resect*.mp. | 246957 |
| 82 | (rod or rods).mp. | 45894 |
| 83 | (screw or screws).mp. | 32905 |
| 84 | ((spine? or spinal*) adj2 fus*).mp. | 20274 |
| 85 | surgeon??.mp. | 128831 |
| 86 | (surgery or surgeries or surgical*).mp. | 1578850 |
| 87 | vertebroplast*.mp. | 2519 |
| 88 | vertebraplast*.mp. | 1 |
| 89 | su.fs. [Surgery floating subheading] | 1690763 |
| 90 | stabili#ation*.mp. | 71237 |
| 91 | instrumentation*.mp. | 24053 |
| 92 | (percutan* adj1 vertebr* adj2 augment*).mp. | 67 |
| 93 | cement*.mp. | 53344 |
| 94 | polymethylmethacrylate??.mp. | 3243 |
| 95 | methylmethacrylate??.mp. | 8031 |
| 96 | skyphoplast*.mp. | 3 |
| 97 | or/36-96 [Surgery & related terms] | 3666306 |
| 98 | 35 and 97 [MESCC + Surgery] | 5452 |
| 99 | exp cohort studies/ | 1520288 |
| 100 | exp prognosis/ | 1238236 |
| 101 | exp morbidity/ | 425360 |
| 102 | exp mortality/ | 309191 |
| 103 | exp survival analysis/ | 213916 |
| 104 | exp models, statistical/ | 310458 |
| 105 | prognos*.mp. | 603120 |
| 106 | predict*.mp. | 1024371 |
| 107 | course*.mp. | 467136 |
| 108 | diagnosed.mp. | 360763 |
| 109 | cohort*.mp. | 387813 |
| 110 | death.mp. | 571243 |
| 111 | exp Risk/ | 933751 |
| 112 | risk*3.mp. | 1740199 |
| 113 | forecast*.mp. | 81576 |
| 114 | (univariat* adj1 analy*).mp. | 34409 |
| 115 | (multivariat* adj1 analy*).mp. | 179844 |
| 116 | (regress* adj1 analy*).mp. | 249720 |
| 117 | meta-regress*.mp. | 3061 |
| 118 | survival.mp. | 858709 |
| 119 | or/99-118 [Prognosis & related terms] | 5776260 |
| 120 | 98 and 119 [ MESCC + Surgery + Prognosis ] | 3370 |
| 121 | animals/ not (animals/ and humans/) | 4191697 |
| 122 | 120 not 121 | 3318 |
| 123 | limit 120 to human | 3318 |
| 124 | 122 or 123 | 3318 |
| 125 | limit 124 to yr="1990 -Current" | 2993 |
| 126 | limit 125 to ("all infant (birth to 23 months)" or "all child (0 to 18 years)" or "newborn infant (birth to 1 month)" or "infant (1 to 23 months)" or "preschool child (2 to 5 years)" or "child (6 to 12 years)" or "adolescent (13 to 18 years)") | 616 |
| 127 | 125 not 126 | 2377 |
| 128 | limit 125 to ("all adult (19 plus years)" or "young adult (19 to 24 years)" or "adult (19 to 44 years)" or "young adult and adult (19-24 and 19-44)" or "middle age (45 to 64 years)" or "middle aged (45 plus years)" or "all aged (65 and over)" or "aged (80 and over)") | 2268 |
| 129 | 127 or 128 | 2780 |
| 130 | remove duplicates from 129 | 2761 |
| 131 | limit 130 to yr="2015 -Current" | 110 |

Ovid MEDLINE(R) In-Process & Other Non-Indexed Citations April 14, 2016

| 1 | Spinal Cord Neoplasms/ | 0 |
| --- | --- | --- |
| 2 | Spinal Neoplasms/ | 0 |
| 3 | Epidural Neoplasms/ | 0 |
| 4 | Epidural Space/ | 0 |
| 5 | ((spine? or spinal) adj3 adenocarcin*).mp. | 4 |
| 6 | ((spine? or spinal) adj3 cancer*).mp. | 36 |
| 7 | ((spine? or spinal) adj3 carcino*).mp. | 17 |
| 8 | ((spine? or spinal) adj3 malign*).mp. | 41 |
| 9 | ((spine? or spinal) adj3 metasta*).mp. | 419 |
| 10 | ((spine? or spinal) adj3 neoplas*).mp. | 78 |
| 11 | ((spine? or spinal) adj3 lesion*).mp. | 513 |
| 12 | ((spine? or spinal) adj3 tumo?r*).mp. | 575 |
| 13 | (epidural adj3 adenocarcin*).mp. | 0 |
| 14 | (epidural adj3 cancer*).mp. | 1 |
| 15 | (epidural adj3 carcin*).mp. | 1 |
| 16 | (epidural adj3 lesion*).mp. | 31 |
| 17 | (epidural adj3 metasta*).mp. | 44 |
| 18 | (epidural adj3 malign*).mp. | 9 |
| 19 | (epidural adj3 neoplas*).mp. | 3 |
| 20 | (epidural adj3 tumo?r*).mp. | 29 |
| 21 | (epidural adj3 lesion*).mp. | 31 |
| 22 | (spinal adj1 cord? adj1 compress*).mp. | 485 |
| 23 | (compress* adj4 myelopath???).mp. | 89 |
| 24 | conus medullaris syndrome?.mp. | 6 |
| 25 | (compress* adj6 spin?? cord?).mp. | 649 |
| 26 | (epidural adj6 compress*).mp. | 116 |
| 27 | or/1-26 [Spinal Cord or Epidural Cancer & related terms] | 2032 |
| 28 | exp Neoplasm Metastasis/ | 0 |
| 29 | sc.fs. [secondary] | 0 |
| 30 | Micrometast*.mp. | 345 |
| 31 | Micro-metast*.mp. | 41 |
| 32 | metasta*.mp. | 36813 |
| 33 | secondary.mp. | 50783 |
| 34 | or/28-33 [Metastasis & related terms] | 86287 |
| 35 | 27 and 34 [Spinal Cord Ca + Metastasis] | 714 |
| 36 | exp Neurosurgical Procedures/ | 0 |
| 37 | Surgery, Computer-Assisted/ | 0 |
| 38 | Surgical Procedures, Minimally Invasive/ | 0 |
| 39 | Surgical Procedures, Minor/ | 0 |
| 40 | Surgical Procedures, Elective/ | 0 |
| 41 | Decompression, Surgical/ | 0 |
| 42 | Neurosurgery/ | 1 |
| 43 | Orthopedics/ | 0 |
| 44 | exp Orthopedic Procedures/ | 0 |
| 45 | exp cementoplasty/ or vertebroplasty/ or kyphoplasty/ | 0 |
| 46 | Foraminotomy/ | 0 |
| 47 | Microsurgery/ | 0 |
| 48 | Metastasectomy/ | 0 |
| 49 | Reconstructive Surgical Procedures/ | 0 |
| 50 | "Prostheses and Implants"/ | 0 |
| 51 | balloon?.mp. | 4041 |
| 52 | (bone adj1 cement*).mp. | 455 |
| 53 | cementoplast*.mp. | 29 |
| 54 | (computer* adj2 aid* adj2 surg*).mp. | 31 |
| 55 | (computer* adj2 assist* adj2 surg*).mp. | 174 |
| 56 | (decompress* adj2 procedur*).mp. | 77 |
| 57 | (decompress* adj2 surg*).mp. | 681 |
| 58 | (elective* adj3 surg*).mp. | 1668 |
| 59 | (excise or excises or excised or excising).mp. | 4638 |
| 60 | excision*.mp. | 9583 |
| 61 | (fixation adj2 devic*).mp. | 209 |
| 62 | fixation?.mp. | 11316 |
| 63 | foraminotom*.mp. | 59 |
| 64 | implant?.mp. | 12538 |
| 65 | kyphoplast*.mp. | 203 |
| 66 | laminectom*.mp. | 721 |
| 67 | laminotom*.mp. | 60 |
| 68 | metastasectom*.mp. | 209 |
| 69 | micro-dissect*.mp. | 59 |
| 70 | microdissect*.mp. | 617 |
| 71 | (minimal* adj2 invasiv*).mp. | 6841 |
| 72 | (minor adj2 surg*).mp. | 244 |
| 73 | neurosurg*.mp. | 3378 |
| 74 | operat*.mp. | 94272 |
| 75 | osteoplast*.mp. | 116 |
| 76 | prosthes*.mp. | 4983 |
| 77 | prosthetic*.mp. | 3364 |
| 78 | (reconstruc* adj2 (spine? or spinal*)).mp. | 54 |
| 79 | (reconstruc* adj2 surg*).mp. | 1886 |
| 80 | remov*3.mp. | 47435 |
| 81 | resect*.mp. | 23815 |
| 82 | (rod or rods).mp. | 6670 |
| 83 | (screw or screws).mp. | 4042 |
| 84 | ((spine? or spinal*) adj2 fus*).mp. | 716 |
| 85 | surgeon??.mp. | 15731 |
| 86 | (surgery or surgeries or surgical*).mp. | 123325 |
| 87 | vertebroplast*.mp. | 353 |
| 88 | vertebraplast*.mp. | 0 |
| 89 | su.fs. [Surgery floating subheading] | 0 |
| 90 | stabili#ation*.mp. | 11069 |
| 91 | instrumentation*.mp. | 4121 |
| 92 | (percutan* adj1 vertebr* adj2 augment*).mp. | 12 |
| 93 | cement*.mp. | 4333 |
| 94 | polymethylmethacrylate??.mp. | 405 |
| 95 | methylmethacrylate??.mp. | 185 |
| 96 | skyphoplast*.mp. | 1 |
| 97 | or/36-96 [Surgery & related terms] | 288354 |
| 98 | 35 and 97 [MESCC + Surgery] | 416 |
| 99 | exp cohort studies/ | 0 |
| 100 | exp prognosis/ | 0 |
| 101 | exp morbidity/ | 0 |
| 102 | exp mortality/ | 0 |
| 103 | exp survival analysis/ | 0 |
| 104 | exp models, statistical/ | 0 |
| 105 | prognos*.mp. | 38984 |
| 106 | predict*.mp. | 140332 |
| 107 | course*.mp. | 35503 |
| 108 | diagnosed.mp. | 44317 |
| 109 | cohort*.mp. | 39806 |
| 110 | death.mp. | 41264 |
| 111 | exp Risk/ | 0 |
| 112 | risk*3.mp. | 154497 |
| 113 | forecast*.mp. | 1678 |
| 114 | (univariat* adj1 analy*).mp. | 3859 |
| 115 | (multivariat* adj1 analy*).mp. | 12416 |
| 116 | (regress* adj1 analy*).mp. | 19615 |
| 117 | meta-regress*.mp. | 611 |
| 118 | survival.mp. | 57085 |
| 119 | or/99-118 [Prognosis & related terms] | 427815 |
| 120 | 98 and 119 [ MESCC + Surgery + Prognosis ] | 218 |
| 121 | limit 120 to yr="2015 -Current" | 112 |

OVID Embase 1974 to 2016 April 14

| 1 | exp spinal cord cancer/ | 2503 |
| --- | --- | --- |
| 2 | exp spine cancer/ | 4058 |
| 3 | epidural space/ | 4661 |
| 4 | ((spine? or spinal) adj3 adenocarcin*).mp. | 33 |
| 5 | ((spine? or spinal) adj3 cancer*).mp. | 1677 |
| 6 | ((spine? or spinal) adj3 carcino*).mp. | 230 |
| 7 | ((spine? or spinal) adj3 malign*).mp. | 727 |
| 8 | ((spine? or spinal) adj3 metasta*).mp. | 7313 |
| 9 | ((spine? or spinal) adj3 neoplas*).mp. | 2301 |
| 10 | ((spine? or spinal) adj3 lesion*).mp. | 12209 |
| 11 | ((spine? or spinal) adj3 tumo?r*).mp. | 18003 |
| 12 | (epidural adj3 adenocarcin*).mp. | 4 |
| 13 | (epidural adj3 cancer*).mp. | 124 |
| 14 | (epidural adj3 carcin*).mp. | 24 |
| 15 | (epidural adj3 lesion*).mp. | 339 |
| 16 | (epidural adj3 metasta*).mp. | 480 |
| 17 | (epidural adj3 malign*).mp. | 85 |
| 18 | (epidural adj3 neoplas*).mp. | 84 |
| 19 | (epidural adj3 tumo?r*).mp. | 448 |
| 20 | (epidural adj3 lesion*).mp. | 339 |
| 21 | (spinal adj1 cord? adj1 compress*).mp. | 14936 |
| 22 | (compress* adj4 myelopath???).mp. | 899 |
| 23 | conus medullaris syndrome?.mp. | 72 |
| 24 | (compress* adj6 spin?? cord?).mp. | 16429 |
| 25 | (epidural adj6 compress*).mp. | 1371 |
| 26 | or/1-25 [Spinal Cord or Epidural Cancer & related terms] | 54446 |
| 27 | exp metastasis/ | 466841 |
| 28 | spinal cord metastasis/ | 2092 |
| 29 | spine metastasis/ | 3370 |
| 30 | Micrometast*.mp. | 9463 |
| 31 | Micro-metast*.mp. | 706 |
| 32 | metasta*.mp. | 641729 |
| 33 | secondary.mp. | 695338 |
| 34 | or/27-33 [Metastasis & related terms] | 1310176 |
| 35 | 26 and 34 [Spinal Cord Ca + Metastasis] | 14506 |
| 36 | balloon?.mp. | 98042 |
| 37 | (bone adj1 cement*).mp. | 13548 |
| 38 | cementoplast*.mp. | 289 |
| 39 | (computer* adj2 aid* adj2 surg*).mp. | 536 |
| 40 | (computer* adj2 assist* adj2 surg*).mp. | 8239 |
| 41 | (decompress* adj2 procedur*).mp. | 1106 |
| 42 | (decompress* adj2 surg*).mp. | 18941 |
| 43 | (elective* adj3 surg*).mp. | 39011 |
| 44 | (excise or excises or excised or excising).mp. | 60418 |
| 45 | excision*.mp. | 145893 |
| 46 | (fixation adj2 devic*).mp. | 5323 |
| 47 | fixation?.mp. | 178067 |
| 48 | foraminotom*.mp. | 699 |
| 49 | inplant?.mp. | 134 |
| 50 | kyphoplast*.mp. | 2539 |
| 51 | laminectom*.mp. | 18394 |
| 52 | laminotom*.mp. | 740 |
| 53 | metastasectom*.mp. | 2459 |
| 54 | micro-dissect*.mp. | 1025 |
| 55 | microdissect*.mp. | 17272 |
| 56 | (minimal* adj2 invasiv*).mp. | 82232 |
| 57 | (minor adj2 surg*).mp. | 5334 |
| 58 | neurosurg*.mp. | 85154 |
| 59 | operat*.mp. | 1205290 |
| 60 | osteoplast*.mp. | 2268 |
| 61 | prosthes*.mp. | 241014 |
| 62 | prosthet*.mp. | 54623 |
| 63 | (reconstruc* adj2 (spine? or spinal*)).mp. | 809 |
| 64 | (reconstruc* adj2 surg*).mp. | 27958 |
| 65 | remov*3.mp. | 624698 |
| 66 | resect*.mp. | 463379 |
| 67 | (rod or rods).mp. | 56126 |
| 68 | (screw or screws).mp. | 44455 |
| 69 | ((spine? or spinal*) adj2 fus*).mp. | 22771 |
| 70 | (surgery or surgeries or surgical*).mp. | 2555026 |
| 71 | surgeon??.mp. | 264419 |
| 72 | vertebroplast*.mp. | 4611 |
| 73 | vertebraplast*.mp. | 6 |
| 74 | su.fs. [Surgery floating subheading] | 1853031 |
| 75 | exp neurosurgery/ | 227942 |
| 76 | neurosurgeon/ | 3017 |
| 77 | computer assisted surgery/ | 7437 |
| 78 | minimally invasive surgery/ | 29062 |
| 79 | minor surgery/ | 1844 |
| 80 | elective surgery/ | 24342 |
| 81 | exp decompression surgery/ | 38897 |
| 82 | exp orthopedic surgery/ | 372380 |
| 83 | orthopedic surgeon/ | 2778 |
| 84 | exp cementoplasty/ | 5148 |
| 85 | exp spinal cord surgery/ | 31155 |
| 86 | exp spine surgery/ | 61507 |
| 87 | microsurgery/ | 24861 |
| 88 | metastasis resection/ | 1833 |
| 89 | bone resection/ | 2375 |
| 90 | plastic surgery/ | 60023 |
| 91 | bone transplantation/ | 17934 |
| 92 | exp orthopedic fixation device/ | 51746 |
| 93 | exp fracture treatment/ | 92825 |
| 94 | exp "prostheses and orthoses"/ | 296441 |
| 95 | implant/ | 41080 |
| 96 | neurological implant/ | 66 |
| 97 | neuroprosthesis/ | 615 |
| 98 | orthopedic implant/ | 711 |
| 99 | stabili#ation*.mp. | 106476 |
| 100 | instrumentation*.mp. | 261115 |
| 101 | (percutan* adj1 verteb* adj2 augment*).mp. | 107 |
| 102 | cement*.mp. | 60597 |
| 103 | polymethylmethacrylate??.mp. | 4011 |
| 104 | methylmethacrylate??.mp. | 2068 |
| 105 | skyphoplast*.mp. | 5 |
| 106 | or/36-105 [Surgery & related terms] | 5210988 |
| 107 | 35 and 106 [Spinal Cord Ca + Metastasis + Surgery] | 8873 |
| 108 | cohort analysis/ | 238206 |
| 109 | exp prognosis/ | 535169 |
| 110 | exp morbidity/ | 274665 |
| 111 | exp mortality/ | 791604 |
| 112 | exp survival/ | 765232 |
| 113 | statistical model/ | 122486 |
| 114 | "prediction and forecasting"/ | 18550 |
| 115 | prognos*.tw. | 623945 |
| 116 | predict*.tw. | 1429300 |
| 117 | course*.tw. | 664866 |
| 118 | diagnosed.tw. | 620022 |
| 119 | cohort*.tw. | 554035 |
| 120 | death.tw. | 709354 |
| 121 | exp Risk/ | 1822022 |
| 122 | risk*3.mp. | 2725933 |
| 123 | forecast*.mp. | 69672 |
| 124 | or/108-123 [Prognosis & related terms] | 6479889 |
| 125 | 107 and 124 [Spinal Cord Ca + Metastasis + Surgery + Prognosis] | 4520 |
| 126 | (exp animals/ or exp animal experimentation/ or nonhuman/) not ((exp animals/ or exp animal experimentation/ or nonhuman/) and exp human/) | 5886780 |
| 127 | 125 not 126 | 4466 |
| 128 | limit 125 to human | 4207 |
| 129 | 127 or 128 | 4466 |
| 130 | limit 129 to embase | 4018 |
| 131 | limit 130 to (embryo <first trimester> or infant <to one year> or child <unspecified age> or preschool child <1 to 6 years> or school child <7 to 12 years> or adolescent <13 to 17 years>) | 520 |
| 132 | 129 not 131 | 3946 |
| 133 | limit 129 to (adult <18 to 64 years> or aged <65+ years>) | 2232 |
| 134 | 132 or 133 | 4225 |
| 135 | limit 134 to (book or book series or conference abstract or conference proceeding or "conference review") | 836 |
| 136 | 134 not 135 | 3389 |
| 137 | remove duplicates from 136 | 3302 |
| 138 | limit 137 to yr="2015 -Current" | 291 |

OVID EBM Reviews - Cochrane Central Register of Controlled Trials February 2016

| 1 | Spinal Cord Neoplasms/ | 32 |
| --- | --- | --- |
| 2 | Spinal Neoplasms/ | 49 |
| 3 | Epidural Neoplasms/ | 1 |
| 4 | Epidural Space/ | 186 |
| 5 | ((spine? or spinal) adj3 adenocarcin*).mp. | 1 |
| 6 | ((spine? or spinal) adj3 cancer*).mp. | 36 |
| 7 | ((spine? or spinal) adj3 carcino*).mp. | 6 |
| 8 | ((spine? or spinal) adj3 malign*).mp. | 9 |
| 9 | ((spine? or spinal) adj3 metasta*).mp. | 82 |
| 10 | ((spine? or spinal) adj3 neoplas*).mp. | 91 |
| 11 | ((spine? or spinal) adj3 lesion*).mp. | 158 |
| 12 | ((spine? or spinal) adj3 tumo?r*).mp. | 65 |
| 13 | (epidural adj3 adenocarcin*).mp. | 2 |
| 14 | (epidural adj3 cancer*).mp. | 19 |
| 15 | (epidural adj3 carcin*).mp. | 3 |
| 16 | (epidural adj3 lesion*).mp. | 3 |
| 17 | (epidural adj3 metasta*).mp. | 6 |
| 18 | (epidural adj3 malign*).mp. | 6 |
| 19 | (epidural adj3 neoplas*).mp. | 4 |
| 20 | (epidural adj3 tumo?r*).mp. | 4 |
| 21 | (epidural adj3 lesion*).mp. | 3 |
| 22 | (spinal adj1 cord? adj1 compress*).mp. | 223 |
| 23 | (compress* adj4 myelopath???).mp. | 13 |
| 24 | conus medullaris syndrome?.mp. | 0 |
| 25 | (compress* adj6 spin?? cord?).mp. | 239 |
| 26 | (epidural adj6 compress*).mp. | 30 |
| 27 | or/1-26 | 807 |
| 28 | exp Neoplasm Metastasis/ | 3537 |
| 29 | sc.fs. [secondary] | 2726 |
| 30 | Micrometast*.mp. | 178 |
| 31 | Micro-metast*.mp. | 17 |
| 32 | metasta*.mp. | 16333 |
| 33 | secondary.mp. | 51676 |
| 34 | spinal cord metastasis/ | 0 |
| 35 | spine metastasis/ | 0 |
| 36 | or/28-35 | 66662 |
| 37 | 27 and 36 | 243 |
| 38 | exp Neurosurgical Procedures/ | 4526 |
| 39 | Surgery, Computer-Assisted/ | 505 |
| 40 | Surgical Procedures, Minimally Invasive/ | 2 |
| 41 | Surgical Procedures, Minor/ | 0 |
| 42 | Surgical Procedures, Elective/ | 0 |
| 43 | Decompression, Surgical/ | 369 |
| 44 | Neurosurgery/ | 72 |
| 45 | Orthopedics/ | 293 |
| 46 | exp Orthopedic Procedures/ | 8748 |
| 47 | exp cementoplasty/ or vertebroplasty/ or kyphoplasty/ | 92 |
| 48 | Foraminotomy/ | 1 |
| 49 | Microsurgery/ | 409 |
| 50 | Metastasectomy/ | 6 |
| 51 | Reconstructive Surgical Procedures/ | 557 |
| 52 | "Prostheses and Implants"/ | 503 |
| 53 | balloon?.mp. | 6556 |
| 54 | (bone adj1 cement*).mp. | 462 |
| 55 | cementoplast*.mp. | 3 |
| 56 | (computer* adj2 aid* adj2 surg*).mp. | 11 |
| 57 | (computer* adj2 assist* adj2 surg*).mp. | 618 |
| 58 | (decompress* adj2 procedur*).mp. | 52 |
| 59 | (decompress* adj2 surg*).mp. | 648 |
| 60 | (elective* adj3 surg*).mp. | 7254 |
| 61 | (excise or excises or excised or excising).mp. | 575 |
| 62 | excision*.mp. | 3152 |
| 63 | (fixation adj2 devic*).mp. | 225 |
| 64 | fixation?.mp. | 4515 |
| 65 | foraminotom*.mp. | 21 |
| 66 | implant?.mp. | 6474 |
| 67 | kyphoplast*.mp. | 102 |
| 68 | laminectom*.mp. | 363 |
| 69 | laminotom*.mp. | 36 |
| 70 | metastasectom*.mp. | 44 |
| 71 | micro-dissect*.mp. | 0 |
| 72 | microdissect*.mp. | 57 |
| 73 | (minimal* adj2 invasiv*).mp. | 2291 |
| 74 | (minor adj2 surg*).mp. | 852 |
| 75 | neurosurg*.mp. | 1541 |
| 76 | operat*.mp. | 45318 |
| 77 | osteoplast*.mp. | 13 |
| 78 | prosthes*.mp. | 8299 |
| 79 | prosthetic*.mp. | 1128 |
| 80 | (reconstruc* adj2 (spine? or spinal*)).mp. | 37 |
| 81 | (reconstruc* adj2 surg*).mp. | 1215 |
| 82 | remov*3.mp. | 14424 |
| 83 | resect*.mp. | 10851 |
| 84 | (rod or rods).mp. | 565 |
| 85 | (screw or screws).mp. | 1619 |
| 86 | ((spine? or spinal*) adj2 fus*).mp. | 1053 |
| 87 | surgeon??.mp. | 7113 |
| 88 | (surgery or surgeries or surgical*).mp. | 102726 |
| 89 | vertebroplast*.mp. | 150 |
| 90 | vertebraplast*.mp. | 0 |
| 91 | su.fs. [Surgery floating subheading] | 45885 |
| 92 | stabili#ation*.mp. | 2903 |
| 93 | instrumentation*.mp. | 1831 |
| 94 | (percutan* adj1 vertebr* adj2 augment*).mp. | 3 |
| 95 | cement*.mp. | 3794 |
| 96 | polymethylmethacrylate??.mp. | 163 |
| 97 | methylmethacrylate??.mp. | 245 |
| 98 | skyphoplast*.mp. | 0 |
| 99 | exp neurosurgery/ [Embase] | 72 |
| 100 | neurosurgeon/ [Embase] | 0 |
| 101 | computer assisted surgery/ [Embase] | 505 |
| 102 | minimally invasive surgery/ [Embase] | 1 |
| 103 | minor surgery/ [Embase] | 0 |
| 104 | elective surgery/ [Embase] | 0 |
| 105 | exp Microvascular Decompression Surgery/ [Embase] | 6 |
| 106 | exp Orthopedics/ [Embase] | 293 |
| 107 | orthopedic surgeon/ [Embase] | 0 |
| 108 | exp cementoplasty/ [Embase] | 92 |
| 109 | microsurgery/ [Embase] | 409 |
| 110 | metastasis resection/ [Embase] | 0 |
| 111 | bone resection/ [Embase] | 0 |
| 112 | plastic surgery/ [Embase] | 103 |
| 113 | bone transplantation/ [Embase] | 673 |
| 114 | exp Orthopedic Fixation Devices/ [Embase] | 1968 |
| 115 | implant/ [Embase] | 0 |
| 116 | neurological implant/ [Embase] | 0 |
| 117 | neuroprosthesis/ [Embase] | 0 |
| 118 | orthopedic implant/ [Embase] | 0 |
| 119 | or/38-118 | 161846 |
| 120 | 37 and 119 | 130 |
| 121 | exp cohort studies/ | 119668 |
| 122 | exp prognosis/ | 113393 |
| 123 | exp morbidity/ | 11816 |
| 124 | exp mortality/ | 10375 |
| 125 | exp survival analysis/ | 15713 |
| 126 | exp models, statistical/ | 12487 |
| 127 | prognos*.mp. | 22717 |
| 128 | predict*.mp. | 49758 |
| 129 | course*.mp. | 32414 |
| 130 | diagnosed.mp. | 17796 |
| 131 | cohort*.mp. | 22247 |
| 132 | death.mp. | 23886 |
| 133 | exp Risk/ | 29259 |
| 134 | risk*3.mp. | 104231 |
| 135 | forecast*.mp. | 622 |
| 136 | (univariat* adj1 analy*).mp. | 1751 |
| 137 | (multivariat* adj1 analy*).mp. | 10780 |
| 138 | (regress* adj1 analy*).mp. | 15878 |
| 139 | meta-regress*.mp. | 63 |
| 140 | survival.mp. | 46223 |
| 141 | cohort analysis/ [Embase] | 6176 |
| 142 | exp survival/ [Embase] | 123 |
| 143 | statistical model/ [Embase] | 985 |
| 144 | "prediction and forecasting"/ [Embase] | 0 |
| 145 | or/121-144 | 343535 |
| 146 | 120 and 145 | 96 |
| 147 | limit 146 to yr="2015 -Current" | 8 |
| 148 | from 147 keep 1-8 | 8 |
|  |  |  |

Interface - EBSCOhost Research Databases 
Search Screen - Advanced Search 
Database - CINAHL with Full Text

Friday, April 15, 2016 4:16:06 PM

| **#** | **Query** | **Limiters/Expanders** | **Results** |
| --- | --- | --- | --- |
| S9 | S5 AND S8 | Limiters - Published Date: 20150101-20171231  Search modes - Boolean/Phrase | 25 |
| S8 | S6 OR S7 | Search modes - Boolean/Phrase | 803,849 |
| S7 | ( risk OR risks OR risked OR risking ) OR forecast* OR univariat* N1 analy* OR multivariat* N1 analy* OR regress* N1 analy* OR meta-regress* OR survival | Search modes - Boolean/Phrase | 467,208 |
| S6 | ( cohort OR cohorts ) OR (MH "Prognosis+") OR (MH "Morbidity+") OR (MH "Mortality+") OR (MH "Survival Analysis+") OR (MH "Models, Statistical") OR prognos* OR predict* OR ( course OR courses ) OR diagnosed OR ( death OR deaths ) OR (MH "Risk Assessment") | Search modes - Boolean/Phrase | 553,891 |
| S5 | S3 AND S4 | Search modes - Boolean/Phrase | 786 |
| S4 | (MH "Neoplasm Metastasis+") OR micrometasta* OR micro-metasta* OR metasta* OR secondary | Search modes - Boolean/Phrase | 78,821 |
| S3 | S1 OR S2 | Search modes - Boolean/Phrase | 2,915 |
| S2 | epidural N3 adenocarcin* OR epidural N3 carcin* OR epidural N3 cancer* OR epidural N3 malig* OR epidural N3 neoplas* OR epidural N3 lesion* OR epidural N3 tumor* OR epidural N3 tumour* OR spin* N1 cord* N1 compress* OR compress* N2 spin* N1 cord* OR epidural N3 compress* OR conus N1 medullaris N1 syndrome* | Search modes - Boolean/Phrase | 1,243 |
| S1 | (MH "Spinal Cord Neoplasms") OR (MH "Spinal Neoplasms") OR ( ((spine or spines or spinal) N3 adenocarcin* ) OR ( ((spine or spines or spinal) N3 cancer* ) OR ( ((spine or spines or spinal) N3 carcin* ) OR ( ((spine or spines or spinal) N3 malig* ) OR ( ((spine or spines or spinal) N3 metasta* ) OR ( ((spine or spines or spinal) N3 neoplas* ) OR ( ((spine or spines or spinal) N3 lesion* ) OR ( ((spine or spines or spinal) N3 tumor* ) OR ( ((spine or spines or spinal) N3 tumour* ) | Search modes - Boolean/Phrase | 1,989 |

Web of Science Core Collection

April 15 2016

| # 31 | [**18**](http://apps.webofknowledge.com.myaccess.library.utoronto.ca/summary.do?product=WOS&doc=1&qid=37&SID=4EXzwP5II2m1P5J3XwN&search_mode=AdvancedSearch&update_back2search_link_param=yes) | (#29 AND #28) *AND***DOCUMENT TYPES:** (Article OR Review)  *Indexes=SCI-EXPANDED Timespan=2015-2016* |
| --- | --- | --- |
| # 30 | [**18**](http://apps.webofknowledge.com.myaccess.library.utoronto.ca/summary.do?product=WOS&doc=1&qid=36&SID=4EXzwP5II2m1P5J3XwN&search_mode=CombineSearches&update_back2search_link_param=yes) | #29 AND #28  *Indexes=SCI-EXPANDED Timespan=2015-2016* |
| # 29 | [**499,880**](http://apps.webofknowledge.com.myaccess.library.utoronto.ca/summary.do?product=WOS&doc=1&qid=35&SID=4EXzwP5II2m1P5J3XwN&search_mode=AdvancedSearch&update_back2search_link_param=yes) | TS= (cohort* OR prognos* OR morbid* OR mortalit* OR predict* OR survival) OR TS= (stastistic* OR course OR courses OR diagnosed OR forecast*) OR TS= ((univariat* N/1 analy*) OR (multivariat* N/1 analys*)) OR TS= ((regress* N/1 analy*) OR metaregress* OR meta-regress*) OR TS= (risk OR risks OR risked OR risking OR death*)  *Indexes=SCI-EXPANDED Timespan=2015-2016* |
| # 28 | [**35**](http://apps.webofknowledge.com.myaccess.library.utoronto.ca/summary.do?product=WOS&doc=1&qid=34&SID=4EXzwP5II2m1P5J3XwN&search_mode=CombineSearches&update_back2search_link_param=yes) | #27 AND #26  *Indexes=SCI-EXPANDED Timespan=2015-2016* |
| # 27 | [**91,503**](http://apps.webofknowledge.com.myaccess.library.utoronto.ca/summary.do?product=WOS&doc=1&qid=33&SID=4EXzwP5II2m1P5J3XwN&search_mode=AdvancedSearch&update_back2search_link_param=yes) | TS=("neoplasm metastas*") OR TS= (micrometast* OR micro-metast*) OR TS=(metasta*) OR TS=(secondary)  *Indexes=SCI-EXPANDED Timespan=2015-2016* |
| # 26 | [**170**](http://apps.webofknowledge.com.myaccess.library.utoronto.ca/summary.do?product=WOS&doc=1&qid=32&SID=4EXzwP5II2m1P5J3XwN&search_mode=CombineSearches&update_back2search_link_param=yes) | #25 OR #12  *Indexes=SCI-EXPANDED Timespan=2015-2016* |
| # 25 | [**6**](http://apps.webofknowledge.com.myaccess.library.utoronto.ca/summary.do?product=WOS&doc=1&qid=31&SID=4EXzwP5II2m1P5J3XwN&search_mode=CombineSearches&update_back2search_link_param=yes) | #24 OR #23 OR #22 OR #21 OR #20 OR #19 OR #18 OR #17 OR #16 OR #15 OR #14 OR #13  *Indexes=SCI-EXPANDED Timespan=2015-2016* |
| # 24 | [**1**](http://apps.webofknowledge.com.myaccess.library.utoronto.ca/summary.do?product=WOS&doc=1&qid=30&SID=4EXzwP5II2m1P5J3XwN&search_mode=AdvancedSearch&update_back2search_link_param=yes) | TS= ((compress* N/3 spinal N/1 cord*))  *Indexes=SCI-EXPANDED Timespan=2015-2016* |
| # 23 | [**2**](http://apps.webofknowledge.com.myaccess.library.utoronto.ca/summary.do?product=WOS&doc=1&qid=29&SID=4EXzwP5II2m1P5J3XwN&search_mode=AdvancedSearch&update_back2search_link_param=yes) | TS= ((epidural N/6 compress*))  *Indexes=SCI-EXPANDED Timespan=2015-2016* |
| # 22 | **0** | TS= ("conus medullaris syndrome*")  *Indexes=SCI-EXPANDED Timespan=2015-2016* |
| # 21 | **0** | TS= ((compress* N/3 myelopath*))  *Indexes=SCI-EXPANDED Timespan=2015-2016* |
| # 20 | [**3**](http://apps.webofknowledge.com.myaccess.library.utoronto.ca/summary.do?product=WOS&doc=1&qid=26&SID=4EXzwP5II2m1P5J3XwN&search_mode=AdvancedSearch&update_back2search_link_param=yes) | TS= ((spinal N/1 cord* N/1 compress*))  *Indexes=SCI-EXPANDED Timespan=2015-2016* |
| # 19 | **0** | TS= ((epidural N/3 tumour*))  *Indexes=SCI-EXPANDED Timespan=2015-2016* |
| # 18 | **0** | TS= ((epidural N/3 tumor*))  *Indexes=SCI-EXPANDED Timespan=2015-2016* |
| # 17 | **0** | TS= ((epidural N/3 neoplas*))  *Indexes=SCI-EXPANDED Timespan=2015-2016* |
| # 16 | **0** | TS= ((epidural N/3 metasta*))  *Indexes=SCI-EXPANDED Timespan=2015-2016* |
| # 15 | **0** | TS= ((epidural N/3 malig*))  *Indexes=SCI-EXPANDED Timespan=2015-2016* |
| # 14 | **0** | TS= ((epidural N/3 carcin*))  *Indexes=SCI-EXPANDED Timespan=2015-2016* |
| # 13 | [**1**](http://apps.webofknowledge.com.myaccess.library.utoronto.ca/summary.do?product=WOS&doc=1&qid=19&SID=4EXzwP5II2m1P5J3XwN&search_mode=AdvancedSearch&update_back2search_link_param=yes) | TS= ((epidural N/3 cancer*))  *Indexes=SCI-EXPANDED Timespan=2015-2016* |
| # 12 | [**167**](http://apps.webofknowledge.com.myaccess.library.utoronto.ca/summary.do?product=WOS&doc=1&qid=18&SID=4EXzwP5II2m1P5J3XwN&search_mode=CombineSearches&update_back2search_link_param=yes) | #11 OR #10 OR #9 OR #8 OR #7 OR #6 OR #5 OR #4 OR #3 OR #2 OR #1  *Indexes=SCI-EXPANDED Timespan=2015-2016* |
| # 11 | **0** | TS= ((epidural N/3 adenocarcin*))  *Indexes=SCI-EXPANDED Timespan=2015-2016* |
| # 10 | [**2**](http://apps.webofknowledge.com.myaccess.library.utoronto.ca/summary.do?product=WOS&doc=1&qid=16&SID=4EXzwP5II2m1P5J3XwN&search_mode=AdvancedSearch&update_back2search_link_param=yes) | TS= (((spine OR spines OR spinal) N/3 tumour*))  *Indexes=SCI-EXPANDED Timespan=2015-2016* |
| # 9 | [**15**](http://apps.webofknowledge.com.myaccess.library.utoronto.ca/summary.do?product=WOS&doc=1&qid=15&SID=4EXzwP5II2m1P5J3XwN&search_mode=AdvancedSearch&update_back2search_link_param=yes) | TS= (((spine OR spines OR spinal) N/3 tumor*))  *Indexes=SCI-EXPANDED Timespan=2015-2016* |
| # 8 | [**19**](http://apps.webofknowledge.com.myaccess.library.utoronto.ca/summary.do?product=WOS&doc=1&qid=14&SID=4EXzwP5II2m1P5J3XwN&search_mode=AdvancedSearch&update_back2search_link_param=yes) | TS= (((spine OR spines OR spinal) N/3 lesion*))  *Indexes=SCI-EXPANDED Timespan=2015-2016* |
| # 7 | [**1**](http://apps.webofknowledge.com.myaccess.library.utoronto.ca/summary.do?product=WOS&doc=1&qid=13&SID=4EXzwP5II2m1P5J3XwN&search_mode=AdvancedSearch&update_back2search_link_param=yes) | TS= (((spine OR spines OR spinal) N/3 neoplas*))  *Indexes=SCI-EXPANDED Timespan=2015-2016* |
| # 6 | [**7**](http://apps.webofknowledge.com.myaccess.library.utoronto.ca/summary.do?product=WOS&doc=1&qid=12&SID=4EXzwP5II2m1P5J3XwN&search_mode=AdvancedSearch&update_back2search_link_param=yes) | TS= (((spine OR spines OR spinal) N/3 metasta*))  *Indexes=SCI-EXPANDED Timespan=2015-2016* |
| # 5 | [**7**](http://apps.webofknowledge.com.myaccess.library.utoronto.ca/summary.do?product=WOS&doc=1&qid=11&SID=4EXzwP5II2m1P5J3XwN&search_mode=AdvancedSearch&update_back2search_link_param=yes) | TS= (((spine OR spines OR spinal) N/3 malig*))  *Indexes=SCI-EXPANDED Timespan=2015-2016* |
| # 4 | [**1**](http://apps.webofknowledge.com.myaccess.library.utoronto.ca/summary.do?product=WOS&doc=1&qid=10&SID=4EXzwP5II2m1P5J3XwN&search_mode=AdvancedSearch&update_back2search_link_param=yes) | TS= (((spine OR spines OR spinal) N/3 carcin*))  *Indexes=SCI-EXPANDED Timespan=2015-2016* |
| # 3 | [**8**](http://apps.webofknowledge.com.myaccess.library.utoronto.ca/summary.do?product=WOS&doc=1&qid=9&SID=4EXzwP5II2m1P5J3XwN&search_mode=AdvancedSearch&update_back2search_link_param=yes) | TS= (((spine OR spines OR spinal) N/3 cancer*))  *Indexes=SCI-EXPANDED Timespan=2015-2016* |
| # 2 | [**1**](http://apps.webofknowledge.com.myaccess.library.utoronto.ca/summary.do?product=WOS&doc=1&qid=8&SID=4EXzwP5II2m1P5J3XwN&search_mode=AdvancedSearch&update_back2search_link_param=yes) | TS= (((spine OR spines OR spinal) N/3 adenocarcin*))  *Indexes=SCI-EXPANDED Timespan=2015-2016* |
| # 1 | [**137**](http://apps.webofknowledge.com.myaccess.library.utoronto.ca/summary.do?product=WOS&doc=1&qid=7&SID=4EXzwP5II2m1P5J3XwN&search_mode=AdvancedSearch&update_back2search_link_param=yes) | TS= ("Spinal Cord Neoplas*" OR "Spinal Neoplas*" OR "Epidural Neoplas*" OR "Epidural Space*")  *Indexes=SCI-EXPANDED Timespan=2015-2016* |

Scopus final string

April 15 2016

( ( ( ( TITLE-ABS-KEY ( spinal cord neoplas* ) OR TITLE-ABS-KEY ( spine neoplas* ) OR TITLE-ABS-KEY ( epidural neoplas* ) OR TITLE-ABS-KEY ( ( ( spine? OR spinal ) W/3 adenocarcin* ) ) OR TITLE-ABS-KEY ( ( ( spine? OR spinal ) W/3 cancer* ) ) OR TITLE-ABS-KEY ( ( ( spine? OR spinal ) W/3 carcin* ) ) OR TITLE-ABS-KEY ( ( ( spine? OR spinal ) W/3 malig* ) ) OR TITLE-ABS-KEY ( ( ( spine? OR spinal ) W/3 metasta* ) ) OR TITLE-ABS-KEY ( ( ( spine? OR spinal ) W/3 lesion* ) ) OR TITLE-ABS-KEY ( ( ( spine? OR spinal ) W/3 neoplas* ) ) OR TITLE-ABS-KEY ( ( ( spine? OR spinal ) W/3 tumor* ) ) OR TITLE-ABS-KEY ( ( ( spine? OR spinal ) W/3 tumour* ) ) OR TITLE-ABS-KEY ( ( epidural W/3 adenocarcin* ) ) OR TITLE-ABS-KEY ( ( epidural W/3 carcin* ) ) OR TITLE-ABS-KEY ( ( epidural W/3 cancer* ) ) OR TITLE-ABS-KEY ( ( epidural W/3 malig* ) ) OR TITLE-ABS-KEY ( ( epidural W/3 metasta* ) ) OR TITLE-ABS-KEY ( ( epidural W/3 neoplas* ) ) OR TITLE-ABS-KEY ( ( epidural W/3 lesion* ) ) OR TITLE-ABS-KEY ( ( epidural W/3 tumor* ) ) OR TITLE-ABS-KEY ( ( epidural W/3 tumour* ) ) OR TITLE-ABS-KEY ( conus W/1 medullaris W/1 syndrome* ) OR TITLE-ABS-KEY ( ( spin* W/4 compress* ) ) OR TITLE-ABS-KEY ( ( epidural W/4 compress* ) ) ) AND PUBYEAR > 1989 ) AND ( ( TITLE-ABS-KEY ( neoplas* W/1 metasta* ) OR TITLE-ABS-KEY ( micrometasta* ) OR TITLE-ABS-KEY ( micro-metasta* ) OR TITLE-ABS-KEY ( metasta* ) OR TITLE-ABS-KEY ( secondary ) ) AND PUBYEAR > 1989 ) ) AND ( ( TITLE-ABS-KEY ( cohort OR cohorts ) OR TITLE-ABS-KEY ( prognos* ) OR TITLE-ABS-KEY ( morbidit* ) OR TITLE-ABS-KEY ( mortalit* ) OR TITLE-ABS-KEY ( death OR deaths ) OR TITLE-ABS-KEY ( survival ) OR TITLE-ABS-KEY ( statistic* W/1 model* ) OR TITLE-ABS-KEY ( predict* ) OR TITLE-ABS-KEY ( course ) OR TITLE-ABS-KEY ( diagnosed ) OR TITLE-ABS-KEY ( risk OR risks OR risked OR risking ) OR TITLE-ABS-KEY ( forecast* ) ) AND PUBYEAR > 1989 ) ) AND ( ( TITLE-ABS-KEY ( neurosurg* ) OR TITLE-ABS-KEY ( surgery OR surgeries OR surgeon* ) OR TITLE-ABS-KEY ( surgical* ) OR TITLE-ABS-KEY ( orthoped* OR orthopaed* ) OR TITLE-ABS-KEY ( decompress* ) OR TITLE-ABS-KEY ( cement* ) OR TITLE-ABS-KEY ( vertebroplast* ) OR TITLE-ABS-KEY ( kyphoplast* ) OR TITLE-ABS-KEY ( foraminotom* ) OR TITLE-ABS-KEY ( microsurger* ) OR TITLE-ABS-KEY ( metastasectom* ) OR TITLE-ABS-KEY ( reconstructiv* ) OR TITLE-ABS-KEY ( prosthe* ) OR TITLE-ABS-KEY ( implant* ) OR TITLE-ABS-KEY ( balloon* ) OR TITLE-ABS-KEY ( excise OR excises OR excised OR excising ) OR TITLE-ABS-KEY ( excision* ) OR TITLE-ABS-KEY ( fixat* ) OR TITLE-ABS-KEY ( laminectom* ) OR TITLE-ABS-KEY ( laminotom* ) OR TITLE-ABS-KEY ( microdissect* OR micro-dissect* ) OR TITLE-ABS-KEY ( minimal* W/1 invasiv* ) OR TITLE-ABS-KEY ( osteoplast* ) OR TITLE-ABS-KEY ( rod OR rods OR screw OR screws OR plate OR plates OR plated OR plating ) OR TITLE-ABS-KEY ( ( spin* W/2 fus* ) ) OR TITLE-ABS-KEY ( vertebroplast* OR vertebraplast* ) OR TITLE-ABS-KEY ( stabilization* OR stabilisation* ) OR TITLE-ABS-KEY ( percutan* W/1 vertebr* W/2 augment* ) OR TITLE-ABS-KEY ( polymethylmethacrylate* ) OR TITLE-ABS-KEY ( methylmethacrylate* ) OR TITLE-ABS-KEY ( skyphoplast* ) ) AND PUBYEAR > 1989 ) AND NOT ( PMID ( 1* OR 2* OR 3* OR 4* OR 5* OR 6* OR 7* OR 8* OR 9* ) ) AND ( LIMIT-TO ( PUBYEAR , 2016 ) OR LIMIT-TO ( PUBYEAR , 2015 ) )
