# Supplementary material for: Symptomatic spinal metastasis: A systematic literature review of the preoperative prognostic factors for survival, neurological, functional and quality of life in surgically treated patients and methodological recommendations for prognostic studies
Source: PLoS One. 2017 Feb 22;12(2):e0171507. doi: 10.1371/journal.pone.0171507 (PMC5321441; doi:10.1371/journal.pone.0171507)
Supplement: S2 Table — (DOCX) [file pone.0171507.s003.docx]

**S2 Table** Different Levels of Evidence for Predictive Studies

| **Class** | **Bias** | **Study design** | **Criteria** |
| --- | --- | --- | --- |
| I | **Low risk**  Study adheres to commonly held tenets of high-quality design, execution, and avoidance of bias | Good-quality cohort* | Prospective design |
|  |  |  | Patients at similar point in the course of their disease or treatment |
|  |  |  | F/U rate of ≥80%† |
|  |  |  | Patients followed long enough for outcomes to occur |
|  |  |  | Accounting for other prognostic factors‡ |
| II | **Moderately low risk**  Study has potential for some bias; does not meet all criteria for Class I but deficiencies not likely to invalidate results or introduce significant bias | Moderate-quality cohort* | Prospective design, with violation of one of the other criteria for good-quality cohort study |
|  |  |  | Retrospective design, meeting all the rest of the criteria in Class I |
| III | **Moderately high risk**  Study has flaws in design and/or execution that increase potential for bias that may invalidate study results | Poor-quality cohort | Prospective design with violation of ≥2 criteria for good-quality cohort |
|  |  | Good-quality case- control or cross- sectional study | Retrospective design with violation of ≥1 criteria for good-quality cohort |
|  |  |  | A good case-control study§ |
|  |  |  | A good cross-sectional study¶ |
| IV | **High risk**  Study has significant potential  for bias, does not include design features geared toward minimizing bias, and/or does not have a comparison group | Poor-quality case- control or cross- sectional study | Other than a good case-control |
|  |  |  | Other than a good cross-sectional study |
|  |  | Case series | Any case series design║ |
| * Cohort studies follow individuals with the exposure of interest over time and monitor for occurrence of the outcome of interest.  † Applies to cohort studies only.  ‡ Authors must consider other factors that might influence patient outcomes.  § A good case-control study must have the all of the following: all incident cases from the defined population during a specified time period, controls that represent the population from which the cases come, exposure that precedes an outcome of interest, and accounting for other prognostic factors.  ¶A good cross-sectional study must have all of the following: a representative sample of the population of interest, an exposure that precedes an outcome of interest (e.g., sex, genetic factor), an accounting for other prognostic factors, and for surveys, at least an 80% return rate.  ║A case series design for prognosis is one in which all the patients in the study have the exposure of interest. Because all the patients have the exposure, risks of an outcome can be calculated only for those with the exposure but cannot be compared with those who do not have the exposure.  **F/U**: follow-up | | | |

From: Dettori JR, Norvell DC, Skelly AC. *Methodology Checklist and Risk of Bias Definitions for Prognostic Studies*: Spectrum Research Standard Operating Procedures for Systematic Review. Tacoma, WA; Spectrum Research, Inc. Evidence-Based Practice Division; 2013.
