# Supplementary material for: Symptomatic spinal metastasis: A systematic literature review of the preoperative prognostic factors for survival, neurological, functional and quality of life in surgically treated patients and methodological recommendations for prognostic studies
Source: PLoS One. 2017 Feb 22;12(2):e0171507. doi: 10.1371/journal.pone.0171507 (PMC5321441; doi:10.1371/journal.pone.0171507)
Supplement: S3 Table — (DOCX) [file pone.0171507.s004.docx]

**S3 Table**: Rating quality of the overall body of evidence

| **Baseline strength** | **Downgrade if** | **Upgrade if** | **Final strength** |
| --- | --- | --- | --- |
| Class I or II | **Risk of bias (study quality)**  -1: serious study limitation in protecting against bias  -2: very serious study limitation in protecting against bias  **Consistency**  -1: either large differences in the magnitude of effect or few studies report a different direction of effect for a particular outcome  -2: either very large differences in the magnitude of effect or many studies report a different direction of effect for a particular outcome  **Directness**  -1: some uncertainty whether predictor is related to outcome of interest  -2: major uncertainty whether predictor is related to outcome of interest  **Precision**  -1: data seriously imprecise so that no clinically useful conclusion can be drawn  -2: data very seriously imprecise so that no clinically useful conclusion can be drawn  **Publication bias**  -1: likely that studies may have been published selectively with regards to the extent to which relevant empirical findings have not been published or are unavailable  -2: very likely that studies may have been published selectively with regards to the extent to which relevant empirical findings have not been published or are unavailable | **Magnitude of effect**  +1: large, i.e. strong association, no plausible confounders  +2: very large, i.e. strong association, no plausible confounders  +1: evidence of a dose response gradient  **All plausible confounding**  +1: would reduce a demonstrated effect  *or*  +1: would suggest a non-genuine effect when results show no effect | **High**  *High confidence that the evidence reflects the true effect. Further research is quite unlikely to change our confidence in the estimate of effect* |
|  |  |  | **Moderate**  *Moderate confidence that the evidence reflects the true effect. Further research may change the estimate and our confidence in the estimate of effect* |
| Class III or IV |  |  | **Low**  *Low confidence that the evidence reflects the true effect. Further research is likely to change the estimate of the effect and our confidence in the estimate of effect* |
|  |  |  | **Very low**  *Either (1) Very low confidence that the evidence reflects the true effect and the true effect is likely to be markedly different from the estimated effect or (2) there is no evidence or it is impossible to estimate an effect* |

Based on Balshem H, Helfand M, Schunemann HJ, Oxman AD, Kunz R, Brozek J, et al. GRADE guidelines: 3. Rating the quality of evidence. J Clin Epidemiol. 2011 Apr;64(4):401-6.
